# A linear Di-coordinate boron radical cation

**DOI:** 10.1038/s41467-022-34900-7

**Published:** 2022-11-17

**Authors:** Yu-Jiang Lin, Wei-Chun Liu, Yi-Hung Liu, Gene-Hsiang Lee, Su-Ying Chien, Ching-Wen Chiu

**Affiliations:** Department of Chemistry, National Taiwan University, No. 1, Section 4, Roosevelt Road, Taipei, 10617 Taiwan

**Keywords:** Chemical bonding, Chemical bonding

## Abstract

The pursuit of di-coordinate boron radical has been continued for more than a half century, and their stabilization and structural characterization remains a challenge. Here we report the isolation and structural characterization of a linear di-coordinate boron radical cation, achieved by stabilizing the two reactive atomic orbitals of the central boron atom by two orthogonal π-donating and π-accepting functionalities. The electron deficient radical cation undergoes facile one-electron reduction to borylene and binds Lewis base to give heteroleptic tri-coordinate boron radical cation. The co-existence of half-filled and empty p orbitals at boron also allows the CO-regulated electron transfer to be explored. As the introduction of CO promotes the electron transfer from a tri-coordinate neutral boron radical to a boron radical cation, the removal of CO under vacuum furnishes the reverse electron transfer from borylene to yield a solution consisting of two boron radicals.

## Abstract (web summary)

The stabilization of low-coordinate boron radicals is challenging, as they tend to form strong chemical bonds. Here the authors report the isolation and structural characterization of a dicoordinate boron radical featuring a π-donating amino and a π-accepting carbene ligand.

## Introduction

**Fig. 1:**
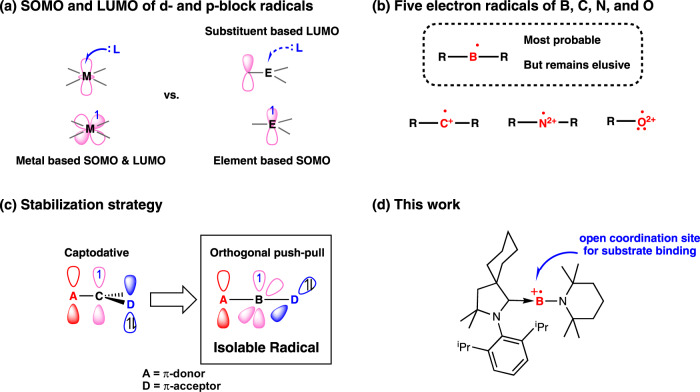
Illustration of the design concept. **a** Frontier MO of d- and p-block radicals. **b** Five electron radicals of 2p elements. **c** Pictorial illustration of captodative and push-pull stabilization effects. **d** The design of boron radical cation.

Electron transfer reaction of low-coordinate high spin metal complexes upon substrate binding is vital for the biological activities of a variety of metalloenzymes. For instance, the activity and function of heme proteins associated with the redox reaction of the system can be modulated by the coordination of gaseous substates like O_2_, NO, and CO to the five-coordinate high spin ferrous center of heme^1^. While the co-existence of the metal-based half-filled (SOMO) and empty (LUMO) frontier molecular orbitals is rather common in transition metal complexes, electron-deficient low-coordinate open-shell main-group compounds are extremely rare and the ligand-regulated electron transfer has not been explored^2–5^. As depicted in Fig. 1a, main-group radicals with no accessible empty valence orbital cannot accept pair of electrons and, thus, frustrate the binding of the substrate at the central atom. Among s- and p-block elements, the synthesis of low-coordinate electron-deficient boron, carbon, nitrogen, and oxygen radicals is exceptionally difficult due to the propensity of these open-shell species to form strong chemical bonds. Considering the overall charge of the molecule and the coordination number at the central atom, neutral di-coordinate boryl radical should be the most probable target in the series (Fig. 1b). However, as tri-coordinate and tetra-coordinate boron radicals^6^ have been extensively explored for applications in organic synthesis^7–11^, polymerization^12–15^, and optoelectronic materials^16–19^, their corresponding five-electron counterparts could only be examined in silico^20^. All attempts in realizing the electron-deficient di-coordinate boron radical from chemical reduction of boranes^21–34^ and borinium ion were unsuccessful^35,36^. Although the heavier Group 13 analogs were readily obtained from reaction of E(I) (E = Ga, In, Tl) precursors with boryllithium or reduction of diborylchloroindium with potassium^2^, the lack of accessible monovalent B(I) precursors and the relatively strong boron-containing chemical bonds should be responsible for the inability to achieve di-coordinate boron radical.

Looking back on the development of carbon radical, the incorporation of both π-donating and π-accepting substituents at a radical center noticeably boosted the lifetime of the radical due to the favorable synergetic stabilization of the unpaired electron. Inspired by the captodative stabilization effect of carbon radical^37–39^, we hypothesized that a linear di-coordinate boron radical could also be accomplished through attaching a captor and a dative group on boron radical (Fig. 1c). However, unlike the captodative effect in which the singly occupied orbital is overlapping with both the donor and the acceptor orbitals, the push-pull stabilizing groups of a linear di-coordinate boron radical are expected to interact with two orthogonal atomic orbitals of boron. As the π-donating substituent could mitigate the extreme Lewis acidity associated with di-coordinate boron atom, the orthogonal π-accepting group could stabilize the unpaired electron through delocalization. Given the fact that the π-acidity of cyclic(alkyl)(amino)carbene (CAAC) has been proven critical in accomplishing isolable main-group radical species^40–42^, we envision that a linear di-coordinate boron radical cation bearing a π-donating bulky amino substituent and a π-acidic CAAC ligand should be an accessible target (Fig. 1d). In this work, we report the synthesis and characterization of a di-coordinate boron radical cation, and demonstrate that the redox event of boron radical cation can be regulated by CO complexation.

## Results and discussion

### Synthesis of di-coordinate boron radical cation

**Fig. 2:**
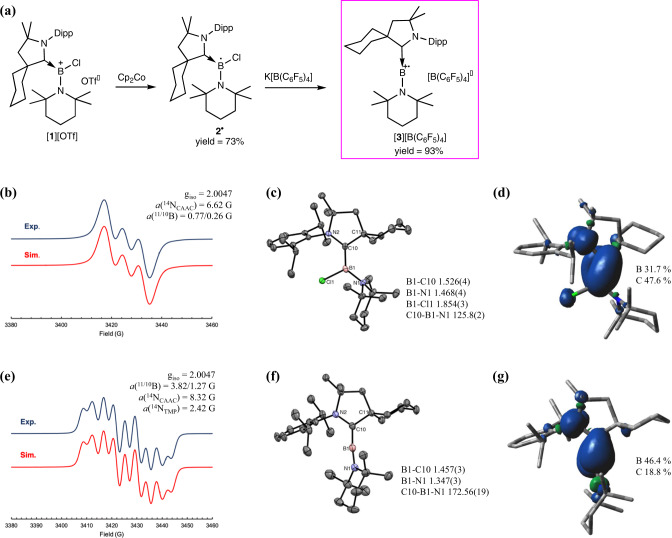
Characterization of 2^•^ and [3][B(C_6_F_5_)_4_]. **a** Synthesis of radical cation [**3**][B(C_6_F_5_)_4_]. EPR spectrum, crystal structure, and calculated spin distribution of **2**^**•**^ (**b**–**d**) and [**3**]^+•^ (**e**–**g**). Dipp = 2,6-diisopropylphenyl, OTf = trifluoromathanesulfonate, CAAC = cyclic(alkyl)(amino)carbene, TMP = 2,2,6,6-tetramethylpiperidinyl.

The CAAC-stabilized amino-chloroborenium [**1**][OTf] obtained from the reaction of equimolar amount of CAAC and TMP-BCl_2_ was reduced in toluene with one equivalent of cobaltocene to give neutral boron radical **2**^**•**^, which was isolated as red solid in 73% yield (Fig. 2a). Radical **2**^**•**^ displays a strong isotropic EPR absorption centered at g_iso_ = 2.0047 with hyperfine coupling to ^14^N and ^11^B/^10^B nuclei of 6.62 G and 0.77/0.26 G, respectively (Fig. 2b). The obtained relatively weak hyperfine coupling to boron is typical for CAAC-stabilized neutral boron radicals^43–47^. Crystalline **2**^**•**^ obtained from slow evaporation of a pentane solution of **2**^**•**^ in a glovebox was analyzed with single crystal X-ray diffraction. As illustrated in Fig. 2c, the presence of a trigonal planar boron center is confirmed by the sum of the E-B-E angles of 360.0°. Structural parameters of **2**^**•**^ are reminiscent of the reported CAAC-stabilized boron radicals. The B–C [1.526(4) Å] and B–N [1.468(4) Å] bond distances are relatively short compared to typical B–C and B–N single bonds due to the delocalization of three electrons in the angular Cπ-Bπ-Nπ orbital. The population of the pπ orbital of boron weakens the B–Cl π-interaction leading to a markedly elongated B–Cl bond [1.854(3) Å], which could be readily cleaved with chloride abstracting agent (vide infra).

**Fig. 3:**
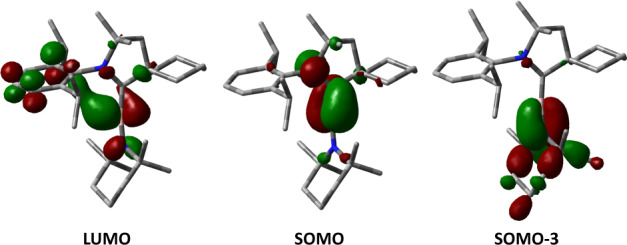
Frontier molecular orbitals of [3]^+•^. LUMO, SOMO, and SOMO-3 respectively represent the B–N π*, B–C π, and B–N π interactions in [**3**]^+•^.

The reaction of **2**^•^ with K[B(C_6_F_5_)_4_] resulted in the formation of a linear di-coordinate boron radical cation [**3**][B(C_6_F_5_)_4_]. A DCM solution consisting of an equimolar amount of **2**^•^ and K[B(C_6_F_5_)_4_] turned gradually from dark red to yellow-greenish to give [**3**][B(C_6_F_5_)_4_] in 93% yield (Fig. 2a). The formation of a new radical species was corroborated by EPR spectroscopy. While the *g*_iso_ value of 2.0047 is identical to that of **2**^•^, a drastic change in the splitting pattern of [**3**]^+•^ is recognized (Fig. 2e). The spectrum can be satisfactorily simulated with one boron nucleus and two inequivalent nitrogen nuclei. Although the obtained hyperfine coupling constants of 3.82 G and 1.27 G to ^11^B (I = 3/2) and ^10^B (I = 3) are relatively small in comparison with triarylborane radical anions^6^, the shift of spin density from CAAC to boron upon chloride abstraction is evident. The unpaired electron of [**3**]^+•^ is also coupled with two inequivalent ^14^N (I = 1) nuclei with hyperfine coupling constants of 8.32 G and 2.42 G, which could be respectively assigned to the nitrogen atom of CAAC and TMP. The considerable contribution of one boron and two nitrogen atoms to the splitting of the EPR signal suggests that the CAAC-B-TMP linkage is preserved in [**3**]^+•^ in the solution. The observed increased delocalization of the unpaired electron to the B-TMP unit is consistent with the enhanced electron deficiency of boron upon chloride abstraction. To verify the existence of a di-coordinate boron radical species, lime green single crystals of [**3**][B(C_6_F_5_)_4_] suitable for X-ray diffraction analysis were obtained by cooling a DCM/pentane solution of [**3**][B(C_6_F_5_)_4_] at −30 °C. As shown in Fig. 3f, a linear di-coordinate boron radical with a C10-B–N1 bond angle of 172.56(19)° is corroborated. The dihedral angle of 67.1° between the planes consisting of the ylidene center of CAAC and the nitrogen atom of TMP is consistent with the presence of two perpendicular π-bonding interactions at the central *sp* hybridized boron atom. The B–C and B–N bond [C10-B1 1.457(3) Å, B1-N1 1.347(3) Å] are shortened by about 6.9 and 12.1 pm with respect to that of **2**^•^, confirming the enhanced π-interactions at the both sides. The solid-state structural features of [**3**]^+•^ are well reflected on its solution EPR spectrum, in which a stronger coupling of the unpaired electron to the B-TMP moiety is recognized.

### DFT calculations of 2^•^ and [3]^+•^

To have a better insight into the bonding situations of **2**^•^ and [**3**]^+•^, density functional theory (DFT) calculations were performed at the UCAM-B3LYP/6-31G**/SMD(CH_2_Cl_2_) level of theory (see Supplementary Methods 1.7). All bond distances and angles of the optimized **2**^•^ and [**3**]^+•^ agree well with those determined experimentally. The assembly of two perpendicular planar substituents at the central boron atom of [**3**]^+•^ in an allene-like linear arrangement is also captured by computation. The discernable enlargement of *a*(^11,10^B) from 0.77/0.26G in **2**^**•**^ to 3.82/1.27G in [**3**]^+•^ can also be elucidated with the spin density distribution calculation. As depicted in Fig. 2d, g, the unpaired electron of **2**^**•**^ and [**3**]^+•^ are mainly localized within the N_CAAC_-C_CAAC_-B unit. However, a drastic shift of the spin density from the ylidene center to boron is observed upon chloride abstraction. The enhanced electron deficiency of boron is responsible for the umpolung of the unpaired electron distribution in the B–C π-bond from C/B = 47.6/31.7 (in **2**^**•**^) to C/B = 18.8/46.4 (in [**3**]^+•^), leading to the observed stronger coupling of the electron spin to the boron nucleus in EPR. Mulliken charge distribution analysis reveals that the positive charge is predominantly residing on the central boron atom of [**3**]^+•^ (+ 0.28, Supplementary Fig. 26), indicating the significant involvement of the borinium ion character to the electronic structure of [**3**]^+•^. The distribution of LUMO, manifesting mainly the 2p orbital of boron, is in line with the electrophilic nature of the boron atom (Fig. 3). Two orthogonal π-bonding interactions of the di-substituted boron radical involving TMP and CAAC are also captured in the frontier molecular orbitals of [**3**]^+•^. As SOMO-3 represents the B = N π-bonding interaction, the share of an unpaired electron between boron and CAAC is depicted in SOMO (Fig. 3).

### Reactions of [3]^+•^

**Fig. 4:**
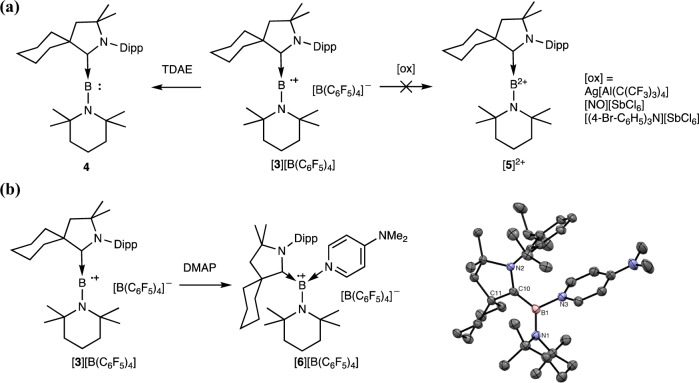
Reactivity studies of [3]^+•^. **a** Reduction and oxidation of [**3**]^+•^. **b** Base coordination reaction and the molecular structure of [**6**]^+•^ Dipp = 2,6-diisopropylphenyl, TDAE = tetrakis(dimethylamino)ethylene, DMAP = 4-dimethylaminopyridine.

With [**3**]^+•^ in hand, we then explored its electrochemical reactions. The cyclic voltammogram of [**3**]^+•^ in DCM features a reversible reduction wave centered at *E*_*1/2*_ = −1.00 V (supplementary Fig. 19), showing that the corresponding CAAC-amino borylene, CAAC-B-TMP (**4**), can be obtained from the one-electron reduction of [**3**]^+•^ (Fig. 4a). As expected, mixing an equimolar amount of tetrakis(dimethylamino)ethylene (TDAE) and [**3**]^+•^ resulted in the clean formation of **4** with the detection of a ^11^B NMR signal at 71.8 ppm comparable to the value reported for CAAC-coordinated aminoborylenes^43,45^. Compared with [**1**]^+^, which exhibits a reversible reduction at *E*_*1/2*_ = −1.18 V with a peak potential difference of 0.50 V (supplementary Fig. 17), the formation of a di-coordinated boron radical cation does not result in a drastic shift of the reduction potential. However, a considerably narrower redox peak separation of 0.27 V is observed for [**3**]^+•^. The obtained not-so-anodic reduction potential and the narrow peak separation of [**3**]^+•^ could be attributed to the strengthened B–C and B–N π-bonding interactions in the linear di-coordinate boron radical cation that mitigate the electron deficiency of the central boron atom and rigidify the central C = B = N core. In contrast to the facile reduction, no identifiable product could be obtained from oxidation of [**3**]^+•^, potentially due to the instability of the resulting di-substituted boron dication, [**5**]^2+^, under the reaction conditions. All attempts in oxidizing [**3**]^+•^ to [**5**]^2+^ with [NO][SbCl_6_], Ag[Al(OC(CF_3_)_3_)_4_], and [(4-Br-C_6_H_4_)_3_N][SbCl_6_] were unsuccessful (Fig. 4a).

According to DFT calculations, there is a substantial borinium ion character to the electronic structure of [**3**]^**+•**^. Thus, we have also examined the reaction of [**3**]^**+•**^ with 4-*N,N*-dimethylaminopyridine (DMAP) to afford a tri-coordinate boron radical cation [**6**]^**+•**^. Upon addition of DMAP, the DCM solution of [**3**]^**+•**^ turned from lime green to violet instantaneously (Fig. 4b). X-ray diffraction analysis on a single crystal of [**6**][B(C_6_F_5_)_4_] obtained from a DCM/pentane solution at –30 °C corroborated the formation of a tri-coordinate boron radical cation, a much less explored boron radical class (Fig. 4b)^46–50^. Compared to the reported homoleptic analogs, the base coordination reaction of [**3**]^**+•**^ represents a straightforward synthetic method to boron radical cations with a heterolepic coordination environment^51,52^. The EPR spectrum of [**6**]^**+•**^ was obtained as a broad signal at –196 °C, which can be satisfactorily simulated with hyperfine coupling to one boron atom (*a*(^11,10^B) = 5.33/1.79G) and three nitrogen atoms (*a*(^14^N_CAAC_) = 8.29 G, *a*(^14^N_TMP_) = 2.63G, and *a*(^14^N_DMAP_) = 1.96G) (supplementary Fig. 23).

### CO-regulated electron transfer

**Fig. 5:**
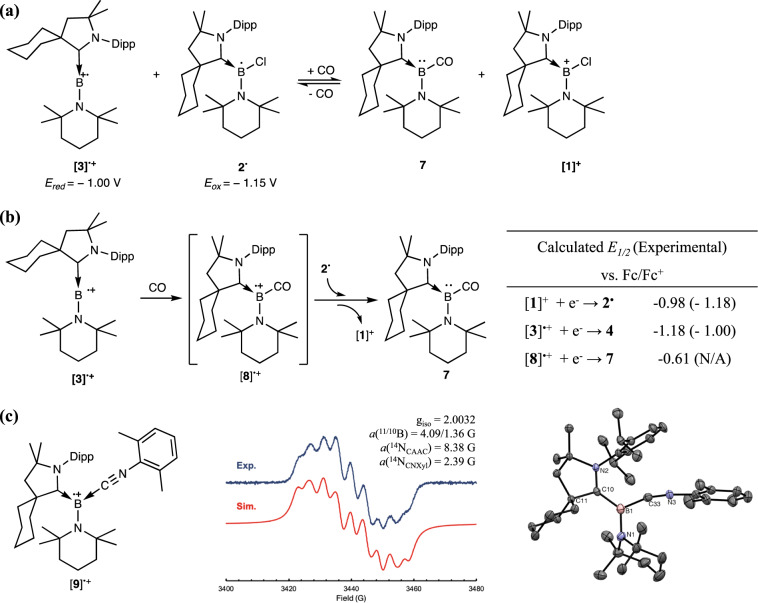
Reaction of [3]^+•^ towards CO and isocyanide. **a** Reaction of 2^•^ and [3]^+•^ with CO. **b** The proposed CO-coordination-initiated electron transfer and the calculated redox potential of relevant species. **c** EPR spectrum and molecular structure of [**9**]^+•^. Dipp = 2,6-diisopropylphenyl, CAAC = cyclic(alkyl)(amino)carbene, CNXyl = 2,6-dimethylphenylisocyanide.

Inspired by the [Mes_2_B]^+^ caused C = O scission of CO_2_^53^, we have also examined the reaction of [**3**]^**+•**^ towards CO_2_ and CO. However, [**3**]^**+•**^ displays no reaction towards CO_2_ and decomposes upon exposure to CO. The inability of [**3**]^**+•**^ in CO_2_ binding could be attributed to the lower Lewis acidity of [**3**]^**+•**^ than [Mes_2_B]^+^, which was corroborated with hydride ion affinity (HIA) calculations (supplementary Table 7)^54^. The presence of an α-amino group and an additional electron at boron significantly diminish the acidity of [**3**]^**+•**^ to afford a HIA of –29.4 kcal/mol, which is 34.2 kcal/mol lower than [Mes_2_B]^+^ (–63.6 kcal/mol). Surprisingly, the corresponding borylene–carbonyl complex (**7**), which could be independently prepared from **4** and CO^43^, was obtained when a 1:1 mixture of **2**^**•**^ and [**3**]^+•^ was allowed to react with CO at room temperature (Fig. 5a). The formation of **7** accompanied with an equimolar amount of chloroborenium ion [**1**]^+^ is intriguing, as **2**^**•**^ with a reduction potential comparable to [**3**]^+•^ cannot reduce the di-coordinate boron radical cation to the corresponding borylene (**4**). As shown in supplementary Fig. 14, no detectable signal of **4** is identified in the NMR spectra of the radical mixture. Upon exposure to CO, two ^11^B NMR signals at −5.6 ppm and 33.0 ppm attributed to **7** and [**1**]^+^ were detected. One interesting property of **7** is its instability under vacuum (supplementary Fig. 16). Upon evacuation, **7** would lose the CO ligand to afford **4** in nearly quantitative yield, implying that the electron transfer between **2**^**•**^ to [**3**]^+•^ can be regulated with CO. We hypothesized that observed CO-regulated electron transfer is due to an anodic shift of the radical cation upon CO-binding and the weak boron–CO interaction in **7**. As the electron deficient nature of carbonyl-boryl radical cation [**8**]^+•^ promotes the electron transfer from **2**^**•**^ to [**8**]^+•^, the reverse transfer of electron from **4** to [**1**]^+^ is observed when **7** decomposes under vacuum to give **4**.

To verify the proposed binding initiated electron transfer, we conducted DFT calculations on the reduction potential of [**1**]^+^, [**3**]^+•^ and [**8**]^+• ^^55^. As summarized in Fig. 5b, the calculated reduction potentials of [**1**]^+^ and [**3**]^+•^ estimated respectively at –0.98 V and –1.18 V are comparable with that determined experimentally. The obtained reversed order is potentially a result of cation-anion interaction in the CV experimental setup. The calculated reduction potential of [**8**]^+•^ is ca. 0.57 V shifted anodic from [**3**]^+•^ at –0.61 V, providing the theoretical support for the enhanced electron deficiency of boron radical cation upon CO coordination. With the calculated reduction potentials, we propose a coordination-initiated electron transfer mechanism for the formation of carbonyl–borylene complex. As depicted in Fig. 5b, the coordination of a π-acidic ligand, CO, to [**3**]^+•^ lowers the reduction potential of the resulting boryl radical cation [**8**]^+•^, which then undergoes subsequent one-electron reduction with **2**^**•**^ to afford **7** and [**1**]^+^.

As all attempts to isolate [**8**]^+•^ were unsuccessful due to the rapid decomposition of the carbonyl-boryl radical cation in the absence of reductant, we decided to mimic the carbonyl binding event with a bulkier isoelectronic analog, 2,6-dimethylphenylisocyanide (xylyl-NC). The addition of xylyl-NC to a DCM solution of [**3**]^+•^ resulted in an instant formation of a dark blue solution, from which dark blue crystals were obtained by adding a few drops of pentane and storing at –30 °C. As demonstrated in Fig. 5c, the binding of isocyanide to the di-coordinate boron radical cation resulted in bending the C_CAAC_–B–N_TMP_ angle from 172.5(2)^o^ in [**3**]^+•^ to 127.1(3)° in [**9**]^+•^. Compared to [**3**]^+•^, the C_*CAAC*_–B bond is elongated from 1.457(3) to 1.536(5) Å due to the competing π-accepting property of the isocyanide group. However, the electron back donation from boron radical to the π* orbital of isocyanide does not lead to appreciable elongation of the C≡N bond, potentially as a consequence of steric repulsion between CAAC and xylyl group. As the consequence of the weak B–C_CN_ interaction, the coordination of xylyl-NC does not lead to a significant shift in redox potential of boron radical cation. Thus, [**9**]^+•^ with a reversible reduction wave at –1.0 V (vs. Fc/Fc^+^ in DCM) cannot be reduced by **2**^•^ to afford the corresponding isocyanide-borylene adduct (**10**).

In conclusion, a di-coordinate boron radical cation, [3]^+•^ stabilized by adjacent orthogonal π-donating and π-accepting groups has been isolated and fully characterized experimentally and computationally. As the crystal structure of [3]^+•^ confirms the linear geometry at boron, DFT calculations identify a significant contribution of borinium ion character to the push-pull stabilized boron radical cation. While the transformation of [3]^+•^ to borylene 4 is readily achieved with a mild reductant, further oxidation to the elusive di-coordinate boron dication [5]^2+^ could not be accomplished. The coordination of [3]^+•^ with DMAP represents a facile synthetic method to heteroleptic tri-coordinate boron radical cation [6]^+•^. Interestingly, the coordination with π-acidic ligand, like CO, triggers subsequent electron transfer from 2^•^ to [8]^+•^ to yield the corresponding carbonyl–borylene complex 7. The removal of CO under vacuum transforms 7 to 4, which then gives an electron to [1]^+^ to afford a solution consisting of two radicals, 2^•^ and [3]^+•^. Such CO-regulated electron transfer could be viewed as a boron-radical based CO-transportation or CO-controlled release of low-coordinate boron radical. Currently, the reaction of [3]^+•^ towards other Lewis bases is under investigation. Fine-tuning the stereoelectronic properties of di-coordinate boron radical cation by replacing CAAC and TMP is also undergoing in this laboratory.

## Methods

### General methods

All the manipulations were conducted using standard Schlenk-line techniques or in a glovebox under nitrogen atmosphere. All solvents were degassed and dried by either molecular sieves (DCM, toluene, hexane), or Na/K (pentane and ether). Cobaltocene (CoCp_2_), lithium diisopropylamide (LDA), tetrakis(dimethylamino)ethylene (TDAE), 4-dimethylaminopyridine (DMAP) and 2,6-dimethylphenylisocyanide (CNXyl) were purchased and used without further purification. Cyclic (alkyl)(amino)carbene (CAAC), 2,2,6,6-tetramethylpiperidinyl boron dichloride (TMP-BCl_2_), potassium tetrakis(pentafluorophenyl) borate (K[B(C_6_F_5_)_4_]) were synthesized according to literature procedures (see Supplementary Methods 1.2). SC-XRD were carried out on Bruker D8 VENTURE at 100 K. NMR spectra were recorded using Bruker AvanceIII-400 (^1^H: 400.2 MHz, ^11^B: 128.4 MHz, ^13^C: 100.6 MHz, ^19^F: 376 MHz). EPR spectra were carried out on either Bruker EMXnano (0.3162 mW) or EMXmicro (21.10 mW). CV spectra were collected on CH Instruments (Model 660D) at a scan rate of 100 mV/s. DFT calculations were carried out with Gaussian 16 program package at the theory level of UCAM-B3LYP/6-31 G(*d,p*) with SMD(DCM) as the solvation model.

### Synthesis of [1][OTf]

A TMP-BCl_2_ (1.85 g, 8.38 mmol) hexane solution was slowly added into a mixture of CAAC (4.00 g, 8.38 mmol) and LiOTf at −78 °C. The resulting solution was then slowly warmed to room temperature and stirred for another 16 h. After the reaction, all volatiles were removed under vacuum and the solid residue was washed by 10 mL of pentane for two times before the addition of 20 mL of dichloromethane. The DCM solution was then filtered and the filtrate was dried to afford [**1**][OTf] as white powder (4.66 g, 84%). X-ray quality crystals were obtained by layering a DCM solution of [**1**][OTf] with pentane. ^1^H NMR (400.2 MHz, CD_2_Cl_2_, ppm): δ 7.53 (t, 1H, *para*-Dipp), 7.39 (d, 2H, *meta*-Dipp), 2.78 (sept, 2H, C*H*(CH_3_)2), 2.46 (s, 2H, C*H*_*2*_), 2.20-1.5 (m, 16H, *H*_*2*_C_Cy,TMP_), 1.63 (s, 6H, C(C*H*_3_)_*2*_), 1.54 (br. s, 6H, NC(C*H*_3_)_2_),1.40 (d, 6H, CH(C*H*_3_)_*2*_), 1.29 (d, 6H, CH(C*H*_3_)_*2*_), 1.12 (br. s, 6H, NC(C*H*_3_)_*2*_). ^11^B NMR (128.4 MHz, CD_2_Cl_2_, ppm): δ 33.1. ^13^C{^1^H} NMR (100.6 MHz, CD_2_Cl_2_, ppm): δ 145.7, 132.3, 131.9, 127.1, 85.3, 62.0, 59.6, 44.0, 40.0, 39.1, 33.3, 33.1, 32.0, 29.4, 28.9, 27.1, 25.8, 24.5, 22.6, 14.9. Anal. Calcd. for C_33_H_53_BClF_3_N_2_O_3_S: C, 59.95; H, 8.08; N, 4.24. Found: C, 59.98; H, 8.02; N, 4.09. ^E^_1/2_ = –1.18 V, peak different 502 mV.

### Synthesis of 2^•^

In a solution CoCp_2_ (1.00 g, 5.29 mmol) in toluene (20 mL), [**1**][OTf] (3.61 g, 5.29 mmol) were added in one portion. After addition, the reaction mixture was stirred over-night, and the solvent was removed in *vacuo*. The product was extracted by 10 mL of pentane to give **2**^**•**^ as reddish powder (1.98 g, 73%). Single crystals were obtained by slow evaporation of a n-pentane solution of **2**^**•**^ at room temperature. Anal. Calcd for C_32_H_53_BClN_2_: C, 75.06; H, 10.43; N, 5.47. Found: C 75.47; H 10.04; N 5.45. *E*_1/2_ = −1.15 V, peak different 430 mV.

### Synthesis of [3][B(C_6_F_5_)_4_]

An equimolar amount of **2**^**•**^ (1.00 g, 1.95 mmol) and K[B(C_6_F_5_)_4_] was allowed to react in 30 mL of DCM at room temperature for 3 h. Afterwards, all insoluble solids were removed by filtration with celite and the filtrate was dried in *vacuo*. The solid residue was then washed with 10 mL of pentane to afford [**3**][B(C_6_F_5_)_4_] as yellow-greenish powder (2.09 g, 93%). X-ray quality crystals were obtained from layering a DCM solution of [**3**][B(C_6_F_5_)_4_] with pentane at –40 ^o^C. Anal. Calcd for C_56_H_53_B_2_F_20_N_2_: C, 58.20; H, 4.62; N, 2.42. Found: C,58.49; H, 4.51; N, 2.31. *E*_1/2_ = −1.00 V, peak different 270 mV.

### Synthesis of 4

An equimolar amount of [**3**][B(C_6_F_5_)_4_] (50.0 mg, 0.043 mmol) and TDAE was mixed in 2 mL of *o*-difluorobenzene. The reaction mixture was stirred for 1 h before the removal of solvent in *vacuo*. Compound **4** was extracted from the solid residue with 3 mL of hexane and obtained as yellow powder (18.4 mg, 89%). ^1^H NMR (400.2 MHz, C_6_D_6_, ppm): δ 7.15-7.10 (m, 3H, *Dipp*), 3.71 (sept, 2H, C*H*(CH_3_)2), 2.33 (m, 2H, *H*_*2*_C_Cy_) 1.94 (s, 2H, C*H*_*2*_), 1.80-1.40 (m, 10H, *H*_*2*_C_Cy,TMP_), 1.38 (s, 6H, C(C*H*_3_)_*2*_), 1.37 (d, 6H, CH(C*H*_3_)_*2*_), 1.31 (d, 6H, CH(C*H*_3_)_*2*_), 1.24 (br. s, 6H, NC(C*H*_3_)_*2*_), 1.15 (br. s, 6H, NC(C*H*_3_)_*2*_). ^11^B NMR (128.4 MHz, C_6_D_6_, ppm): δ 71.8. ^13^C{^1^H} NMR (100.6 MHz, C_6_D_6_, ppm): δ 151.7, 142.5, 126.5, 123.9, 63.8, 55.7, 53.2, 47.5, 44.4, 38.3, 35.3, 31.5, 27.8, 27.5, 26.9, 26.3, 25.9, 25.5, 17.2.

### Synthesis of [6][B(C_6_F_5_)_4_]

DCM (10 mL) was added to the mixture consisting of [**3]**[B(C_6_F_5_)_4_] (50 mg, 0.043 mmol) and DMAP (5.5 mg, 0.043 mmol) at ambient temperature. After stirring for 1 h, solvent was removed with vacuum and the solid residue was washed by 10 mL of pentane to afford [**6**][B(C_6_F_5_)_4_] as violet powder, which was further purified with re-crystallization from DCM/pentane solution (37.7 mg, 68%). Single crystals suitable for X-ray diffraction analysis were obtained from layering a DCM solution of [**6**][B(C_6_F_5_)_4_] with pentane at –40 ^o^C. Anal. Calcd for C_63_H_63_B_2_F_20_N_4_: C, 59.22; H, 4.97; N, 4.38. Found: C, 58.91; H, 4.95; N, 4.05.

### Synthesis of 7

DCM (0.5 mL) was added to the J-Young’s NMR tube consisting of **2**^**•**^ (17.7 mg, 0.035 mmol) and [**3**][B(C_6_F_5_)_4_] (40.0 mg, 0.035 mmol) at ambient temperature. The solution was degassed with three freeze-pump-thaw cycles. Then, the NMR tube was then connected though a tygon tubing to a CO-filled 100 mL Schlenk flask at ambient pressure. After the exposure to CO atmosphere to for 18 h, all volatiles were removed in *vacuo*. **7** was obtained from the reaction mixture by hexane extraction and isolated as reddish powder. ^1^H NMR (400.2 MHz, CD_2_Cl_2_, ppm): δ 7.36 (t, 1H, *para*-Dipp), 7.22 (d, 2H, *meta*-Dipp), 2.96 (sept, 2H, C*H*(CH_3_)2), 2.71 (m, 2H, *H*_*2*_C_Cy_), 2.10 (s, 2H, C*H*_*2*_), 1.90-1.60 (m, 8H, *H*_*2*_C_Cy_), 1.50-1.40 (m, 6H, *H*_*2*_C_TMP_), 1.31 (s, 6H, C(C*H*_3_)_*2*_), 1.30 (d, 6H, CH(C*H*_3_)_*2*_), 1.25 (d, 6H, CH(C*H*_3_)_*2*_), 1.21 (br. s, 6H, NC(C*H*_3_)_*2*_), 1.00 (br. s, 6H, NC(C*H*_3_)_*2*_). ^11^B NMR (128.4 MHz, CD_2_Cl_2_, ppm): δ −5.6. ^13^C{^1^H} NMR (100.6 MHz, C_6_D_6_, ppm): δ 229.4 (m, B*C*O), 202.5 (m, *C*_CAAC_B),149.8, 134.3, 130.2, 126.0, 67.6, 55.4, 54.0, 48.2, 42.7, 37.7, 36.2, 32.21, 30.76, 28.2, 28.1, 27.2, 26.3, 25.2, 23.6, 19.0.

### Synthesis of [9][B(C_6_F_5_)_4_]

DCM (10 mL) was added to the mixture consisting of [**3**][B(C_6_F_5_)_4_] (100 mg, 0.086 mmol) and CNXyl (12.5 mg, 0.095 mmol) at ambient temperature. After stirring for 1 h, the reaction solution was concentrated to about 2 mL before the addition of 20 mL of pentane. The precipitates were collected and dried to afford [**9**][B(C_6_F_5_)_4_] as violet powder (91 mg, 81%). X-ray quality crystals were obtained from layering a DCM solution of [**9**][B(C_6_F_5_)_4_] with pentane at –40 ^o^C. Anal. Calcd for C_65_H_62_B_2_F_20_N_3_•CH_2_Cl_2_ + 0.5C_5_H_12_: C, 58.44; H, 5.01; N, 2.98. Found: C, 59.18; H, 4.80; N, 3.07. *E*_1/2_ = –1.02 V, peak different 103 mV.

## Supplementary information

Supplementary Information

## Data Availability

Experimental details on the synthesis and characterization of all reported compounds can be found in the supporting information. Data supporting the formation of all isolated compounds include NMR spectra, EPR spectra, cyclic voltammogram, and DFT calculations. Crystallographic data of **2**^•^, [**3**][B(C_6_F_5_)_4_], [**6**][B(C_6_F_5_)_4_], and [**9**][B(C_6_F_5_)_4_] have been deposited to CCDC database with the deposition number of CCDC 2167148, 2167149, 2167150, and 2167151.
