# Supplementary material for: A linear Di-coordinate boron radical cation
Source: Nat Commun. 2022 Nov 17;13:7051. doi: 10.1038/s41467-022-34900-7 (PMC9671878; doi:10.1038/s41467-022-34900-7)
Supplement: Supplementary file 1 — Supplementary Information [file 41467_2022_34900_MOESM1_ESM.pdf]

Supplementary Information

for

A Linear Di-coordinate Boron Radical Cation

Yu-Jiang Lin[1‡], Wei-Chun Liu[1‡], Yi-Hung Liu[1], Gene-Hsiang Lee[1], Su-Ying Chien[1] and Ching-Wen Chiu[1]*

[1]Department of Chemistry, National Taiwan University, No. 1, Section 4, Roosevelt Road, Taipei 10617, Taiwan
‡ = These authors contributed equally.
* = Corresponding author, email: cwchiu@ntu.edu.tw

Table of Contents

1. Supplementary Methods 2

1.1. General Consideration 2

1.2. Synthetic Methods 3

1.3. Structural Determination 6

1.4. NMR spectra 15

1.5. Electrochemical Measurement 22

1.6. EPR Spectra and Simulations 26

1.7. Computational Details 28

2. Supplementary References 61

1. Supplementary Methods

1.1. General Consideration

All the manipulations were carried out under nitrogen atmosphere by using standard Schlenk-line techniques or in a glove-box. Toluene, hexane and dichloromethane were purified by with a molecular sieves packed solvent purification system. Pentane and diethyl ether were dried from Na/K-benzophenone ketyl and distilled under nitrogen. Cobaltocene ($CoCp_2$), lithium diisopropylamide (LDA), tetrakis(dimethylamino)ethylene (TDAE), *4*-dimethylaminopyridine (DMAP) and 2,6-dimethylphenylisocyanide (CNXyl) were purchased and used without further purification. Cyclic (alkyl)(amino)carbene (CAAC), *2,2,6,6*-tetramethylpiperidine boron dichloride (TMP-BCl_2), potassium tetrakis(pentafluorophenyl) borate ($K[B(C_6F_5)_4]$) were synthesized according to literature procedures.[1-3] NMR spectra were recorded using Bruker AvanceⅢ-400 (^{1}H: 400.2 MHz, ^{11}B: 128.4 MHz, ^{13}C: 100.6 MHz, ^{19}F: 376 MHz). Chemical Shifts (δ) are given in ppm and are referenced to the signals of the residual solvents (1H, 13C) or external $BF_3·OEt_2$ (^{11}B). Elemental analysis were performed on a Heraeus varioⅢ-NCH elemental analyzer.

1.2. Synthetic Methods

Synthesis of CAAC

In a pre-cooled (0 °C) solution of *N*-2,6-diisopropylphenyl-*C*-cyclohexyl aldimine in Et_2O, an equimolar amount of LDA (2.0 M in THF/heptane) was slowly added. After stirring for 3 hours at room temperature, the mixture was dried under reduced pressure. The residue was re-dissolved in Et_2O and cooled to -78 °C, and then an equimolar amount of 3-chloro-2-methylpropene was slowly added. After stirring for 12 hours at room temperature, all volatiles were evaporated under reduced pressure, and the residue was extracted with hexane and water. The collected organic layer was dried with $MgSO_4$ and the solvent was removed in *vacuo* to afford the alkenyl aldimine (yield 79%). Without further purification, the corresponding alkenyl aldimine was dissolved in CH_3CN, and two equivalent of HCl (2.0 M in Et_2O) was slowly added. After stirring for 20 hours at 50 °C, the mixture was dried in *vacuo*, and the residue was reprecipitated with DCM and Et_2O affording white powder (CAAC•HCl). The solid was dissolved in water and an equimolar amount of HOTf was slowly added. After stirring for 30 mins, white precipitate (CAAC•HOTf) was collected and washed by cold Et_2O (yield 66%). The CAAC•HOTf was dried at 60 °C in *vacuo* for 12 hours, and was stored in an N_2-filled glove box. CAAC was generated by deprotonating the corresponding iminium salt with LiHMDS in Et_2O. The mixture of CAAC and LiOTf could be utilized directly after removing the solvent in *vacuo*.

Synthesis of TMP-BCl_2

2,2,6,6-Tetramethylpiperidine dissolved in hexane was cooled to 0 °C. Then, an equimolar amount of n-butyllithium (2.5 M in hexane) was slowly added. After stirring for 3 hours at 0 °C, the mixture was cooled to -78 °C, and an equimolar amount of BCl_3 (1.0 M in hexane) was slowly added. After addition, the resulting mixture was allowed to stir for 12 hours at room temperature. The mixture was filtered and the filtrate was dried in vacuo to yield TMP-BCl_2 as a pale yellow oil (yield 92%).

Synthesis of $K[B(C_6F_5)_4]$

Bromopentafluorobenzene was dissolved in Et_2O and was cooled to -78 °C. An equimolar amount of n-butyllithium (2.5 M in hexane) was added dropwise over 2 hours and the mixture was stirred for an hour at -78 °C. A quarter molar amount of BCl_3 (1.0 M in hexane) was added dropwise and was stirred for an hour at -78 °C. The mixture was removed from the cooling bath and was stirred for 16 hours at room temperature before the addition of KCl. Then water was added and the mixture was stirred for 1 hour. The organic layer was washed by water several times and the collected organic layer was dried at reduced pressure. The residue was heated at 160 °C in vacuo for 20 hours to give $K[B(C_6F_5)_4]$ a pale brownish powder (yield 80%). ***Caution: The lithiation of bromopentafluorobenzene could be explosive due to the formation of highly reactive benzyne. Thus, the experiment must be carried out with a highly diluted solution at -78 °C, and the addition of n-butyllithium must be very slow.***

Synthesis of [1][OTf]

A TMP-BCl_2 (1.85 g, 8.38 mmol) hexane solution was slowly added into a mixture of CAAC (4.00 g, 8.38 mmol) and LiOTf at -78 °C. The resulting solution was then slowly warmed to room temperature and stirred for another 16 hours. After the reaction, all volatiles were removed under vacuum and the solid residue was washed by 10 mL of pentane for two times before the addition

of 20 mL of dichloromethane. The DCM solution was then filtered and the filtrate was dried to afford [**1**][OTf] as white powder (4.66 g, 84%). X-ray quality crystals were obtained by layering a DCM solution of [**1**][OTf] with pentane. ^{1}H NMR (400.2 MHz, CD_2Cl_2, ppm): δ 7.53 (t, 1H, *para*-Dipp), 7.39 (d, 2H, *meta*-Dipp), 2.78 (sept, 2H, C*H*(CH_3)2), 2.46 (s, 2H, C*H*$_2$), 2.20-1.5 (m, 16H, $H_2C_{Cy,TMP}$), 1.63 (s, 6H, C(CH_3)$_2$), 1.54 (br. s, 6H, NC(CH_3)$_2$),1.40 (d, 6H, CH(CH_3)$_2$), 1.29 (d, 6H, CH(CH_3)$_2$), 1.12 (br. s, 6H, NC(CH_3)$_2$). ^{11}B NMR (128.4 MHz, CD_2Cl_2, ppm): δ 33.1. $^{13}C\{^{1}H\}$ NMR (100.6 MHz, CD_2Cl_2, ppm): δ 145.7, 132.3, 131.9, 127.1, 85.3, 62.0, 59.6, 44.0, 40.0, 39.1, 33.3, 33.1, 32.0, 29.4, 28.9, 27.1, 25.8, 24.5, 22.6, 14.9. Anal. Calcd. for $C_{33}H_{53}BClF_3N_2O_3S$: C, 59.95; H, 8.08; N, 4.24. Found: C, 59.98; H, 8.02; N, 4.09.
$E_{1/2}$ = -1.18 V, peak different 502 mV.

Synthesis of 2•
In a solution $CoCp_2$ (1.00 g, 5.29mmol) in toluene (20 mL), [**1**][OTf] (3.61 g, 5.29 mmol) were added in one portion. After addition, the reaction mixture was stirred over-night, and the solvent was removed in *vacuo*. The product was extracted by 10 mL of pentane to give **2•** as reddish powder (1.98 g, 73%). Single crystals were obtained by slow evaporation of a n-pentane solution of **2•** at room temperature. Anal. Calcd for $C_{32}H_{53}BClN_2$: C, 75.06; H, 10.43; N, 5.47. Found: C 75.47; H 10.04; N 5.45.
$E_{1/2}$ = -1.15 V, peak different 430 mV.

Synthesis of [3][B(C_6F_5)$_4$]
An equimolar amount of **2•** (1.00 g, 1.95 mmol) and K[B(C_6F_5)$_4$] was allowed to react in 30 mL of DCM at room temperature for 3 hours. Afterwards, all insoluble solids were removed by filtration with celite and the filtrate was dried in *vacuo*. The solid residue was then washed with 10 mL of pentane to afford [**3**][B(C_6F_5)$_4$] as yellow-greenish powder (2.09 g, 93%). X-ray quality crystals were obtained from layering a DCM solution of [**3**][B(C_6F_5)$_4$] with pentane at – 40 °C. Anal. Calcd for $C_{56}H_{53}B_2F_{20}N_2$: C, 58.20; H, 4.62; N, 2.42. Found: C,58.49; H, 4.51; N, 2.31.
$E_{1/2}$ = -1.00 V, peak different 270 mV.

Synthesis of 4
An equimolar amount of [**3**][B(C_6F_5)$_4$] (50.0 mg, 0.043 mmol) and TDAE was mixed in 2 mL of *o*-difluorobenzene. The reaction mixture was stirred for 1 hour before the removal of solvent in *vacuo*. Compound **4** was extracted from the solid residue with 3 mL of hexane and obtained as yellow powder (18.4 mg, 89%). ^{1}H NMR (400.2 MHz, C_6D_6, ppm): δ 7.15-7.10 (m, 3H, *Dipp*), 3.71 (sept, 2H, C*H*(CH_3)2), 2.33 (m, 2H, H_2C_{Cy}) 1.94 (s, 2H, C*H*$_2$), 1.80-1.40 (m, 10H, $H_2C_{Cy,TMP}$), 1.38 (s, 6H, C(CH_3)$_2$), 1.37 (d, 6H, CH(CH_3)$_2$), 1.31 (d, 6H, CH(CH_3)$_2$), 1.24 (br. s, 6H, NC(CH_3)$_2$), 1.15 (br. s, 6H, NC(CH_3)$_2$). ^{11}B NMR (128.4 MHz, C_6D_6, ppm): δ 71.8. $^{13}C\{^{1}H\}$ NMR (100.6 MHz, C_6D_6, ppm): δ 151.7, 142.5, 126.5, 123.9, 63.8, 55.7, 53.2, 47.5, 44.4, 38.3, 35.3, 31.5, 27.8, 27.5, 26.9, 26.3, 25.9, 25.5, 17.2.

Synthesis of [6][B(C_6F_5)$_4$]
DCM (10 mL) was added to the mixture consisting of [**3**][B(C_6F_5)$_4$] (50 mg, 0.043 mmol) and DMAP (5.5 mg, 0.043 mmol) at ambient temperature. After stirring for 1 hour, solvent was removed with vacuum and the solid residue was washed by 10 mL of pentane to afford [**6**][B(C_6F_5)$_4$] as violet powder, which was further purified with re-crystallization from DCM/pentane solution (37.7 mg, 68%). Single crystals suitable for X-ray diffraction analysis were

obtained from layering a DCM solution of [**6**][$B(C_6F_5)_4$] with pentane at – 40°C. Anal. Calcd for $C_{63}H_{63}B_2F_{20}N_4$: C, 59.22; H, 4.97; N, 4.38. Found: C, 58.91; H, 4.95; N, 4.05.

Synthesis of 7

DCM (0.5 mL) was added to the J-Young's NMR tube consisting of **2**$^•$ (17.7 mg, 0.035 mmol) and [**3**][$B(C_6F_5)_4$] (40.0 mg, 0.035 mmol) at ambient temperature. The solution was de-gassed with three freeze-pump-thaw cycles. Then, the NMR tube was then connected though a tygon tubing to a CO-filled 100 mL Schlenk flask at ambient pressure. After the exposure to CO atmosphere to for 18 hours, all volatiles were removed in *vacuo*. **7** was obtained from the reaction mixture by hexane extraction and isolated as reddish powder. ^{1}H NMR (400.2 MHz, CD_2Cl_2, ppm): δ 7.36 (t, 1H, *para*-Dipp), 7.22 (d, 2H, *meta*-Dipp), 2.96 (sept, 2H, C*H*(CH_3)2), 2.71 (m, 2H, H_2C_{Cy}), 2.10 (s, 2H, CH_2), 1.90-1.60 (m, 8H, H_2C_{Cy}), 1.50-1.40 (m, 6H, H_2C_{TMP}), 1.31 (s, 6H, C(CH_3)$_2$), 1.30 (d, 6H, CH(CH_3)$_2$), 1.25 (d, 6H, CH(CH_3)$_2$), 1.21 (br. s, 6H, NC(CH_3)$_2$), 1.00 (br. s, 6H, NC(CH_3)$_2$). ^{11}B NMR (128.4 MHz, CD_2Cl_2, ppm): δ -5.6. ^{13}C{^{1}H} NMR (100.6 MHz, C_6D_6, ppm): δ 229.4 (m, B*C*O), 202.5 (m, C_{CAAC}B),149.8, 134.3, 130.2, 126.0, 67.6, 55.4, 54.0, 48.2, 42.7, 37.7, 36.2, 32.21, 30.76, 28.2, 28.1, 27.2, 26.3, 25.2, 23.6, 19.0.

Synthesis of [9][$B(C_6F_5)_4$]

DCM (10 mL) was added to the mixture consisting of [**3**][$B(C_6F_5)_4$] (100 mg, 0.086 mmol) and CNXyl (12.5 mg, 0.095 mmol) at ambient temperature. After stirring for 1 hour, the reaction solution was concentrated to about 2 mL before the addition of 20 mL of pentane. The precipitates were collected and dried to afford [**9**][$B(C_6F_5)_4$] as violet powder (91 mg, 81%). X-ray quality crystals were obtained from layering a DCM solution of [**9**][$B(C_6F_5)_4$] with pentane at – 40 °C. Anal. Calcd for $C_{65}H_{62}B_2F_{20}N_3$•CH_2Cl_2+0.5C_5H_{12}: C, 58.44; H, 5.01; N, 2.98. Found: C, 59.18; H, 4.80; N, 3.07.

$E_{1/2}$ = -1.02 V, peak different 103 mV.

1.3. Structural Determination

The crystal data were collected on a Bruker D8 VENTURE diffractometer at 100(2) K. Data integration and reduction were undertaken with Apex4. Absorption corrections were applied to the data using Multi-scan (Apex4; bruker, 2022). Structures were solved by direct methods using SHELX-2018/3 than refined and extended with SHELX-2018/3. Non-hydrogen atoms were refined anisotropically. Carbon-bound hydrogen atoms were included in idealized positions and refined using a riding model. The molecule was modeled using standard crystallographic methods including constraints, restraints and rigid bodies where necessary. Crystallographic data along with data collection and refinement details were provided in the table below. Crystallographic data of **2**$^{\bullet}$, [**3**][B(C_6F_5)$_4$], [**6**][B(C_6F_5)$_4$], and [**9**][B(C_6F_5)$_4$] were deposited at the Cambridge Crystallographic Data Center with deposition numbers CCDC 2167148, 2167149, 2167150, and 2167151.

The structure of [**9**][B(C_6F_5)$_4$] contains additional pentane and DCM of solvent accessible voids. Due to solvent is multicomponent disordered that modeled might not work satisfactorily even with restraints and the residual electron density peaks are not arranged in an interpretable pattern. The structure factors were instead augmented via reverse Fourier transform methods using the PLATON/SQUEEZE routine (A.L. Spek. Acta Cryst. (2015) C71, 9-18) The resultant FAB file containing the structure factor contribution from the electron content of the void space was used in together with the original hkl file in the further refinement. (The FAB file with details of the Squeeze results is appended to this cif file). The Squeeze procedure corrected for 120 electrons within the solvent accessible voids.

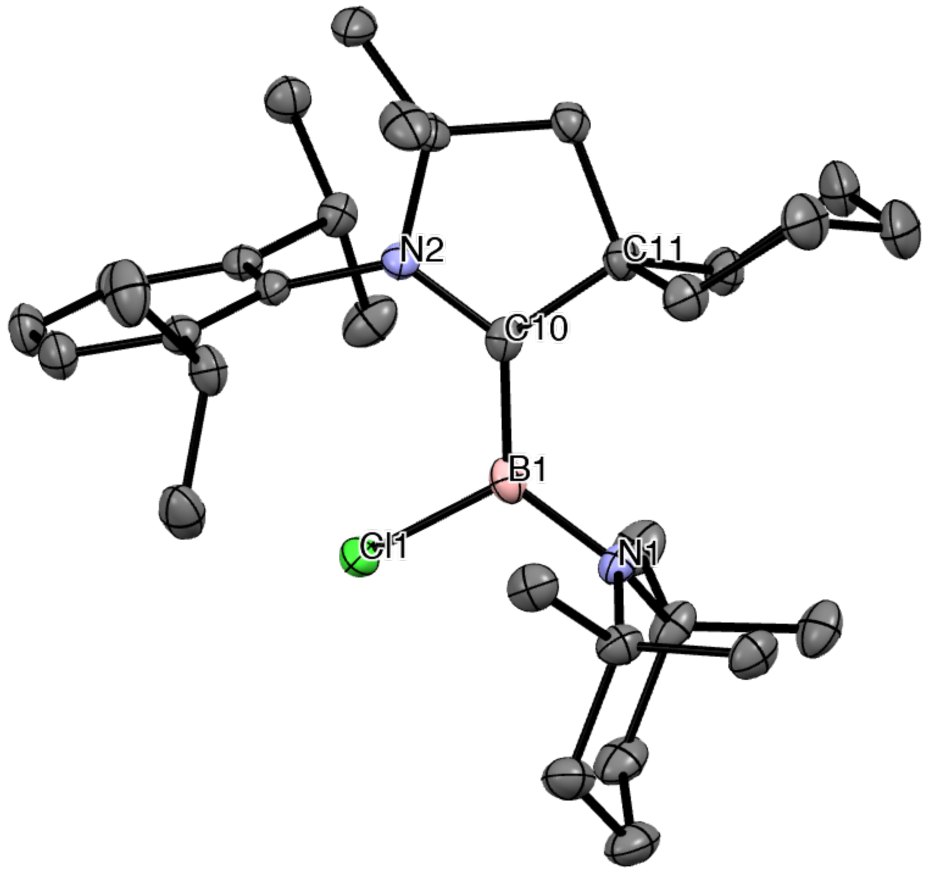

Supplementary Fig. 1. Crystal structure of **2**$^{\bullet}$. Hydrogen atoms are omitted for clarity. Thermal ellipsoids are set at 50% probability. Selected bond distances [Å], bond angles [°]: N2-C10 1.376(3), C10-C11 1.541(4), C10-B1 1.526(4), B1-N1 1.468(4), B1-Cl1 1.854(3), N2-C10-C11 107.7(2), N2-C10-B1 128.4(2), C11-C10-B1 123.9(2), C10-B1-Cl1 117.9(2), C10-B1-N1 125.8(2), Cl1-B1-N1 116.3(2).

Supplementary Table 1. Crystal data and experimental details for **2•**.

CCDC Number	2167148	
Empirical formula	C32 H53 B Cl N2	
Formula weight	512.02	
Crystal system	Monoclinic	
Space group	*C2*	
Unit cell dimensions	a = 32.5544(13) Å	α = 90°.
	b = 9.3874(4) Å	β = 108.9456(15)°.
	c = 20.6472(8) Å	γ = 90°.
Volume	5968.0(4) $Å^3$	
Z	8	
F(000)	2248	
Density (calculated)	1.140 Mg/m^3	
Wavelength	0.71073 Å	
Cell parameters reflections used	9613	
Theta range for Cell parameters	2.41 to 27.43°.	
Absorption coefficient	0.151 mm^{-1}	
Temperature	100(2) K	
Crystal size	0.250 x 0.200 x 0.100 mm^3	
**Data collection and refinement**		
Diffractometer	Bruker D8 VENTURE	
Absorption correction	Semi-empirical from equivalents	
Max. and min. transmission	1.0000 and 0.9335	
No. of measured reflections	75599	
No. of independent reflections	13647 [R(int) = 0.0565]	
No. of observed [I>2igma(I)]	12287	
Completeness to theta = 25.242°	99.9 %	
Theta range for data collection	1.393 to 27.499°.	
Final R indices [I>2sigma(I)]	R1 = 0.0456, wR2 = 0.1095	
R indices (all data)	R1 = 0.0520, wR2 = 0.1148	
Goodness-of-fit on F^2	1.020	
No. of reflections	13647	
No. of parameters	669	
No. of restraints	1	
Absolute structure parameter	0.002(19)	
Largest diff. peak and hole	0.334 and -0.438 e.$Å^{-3}$	

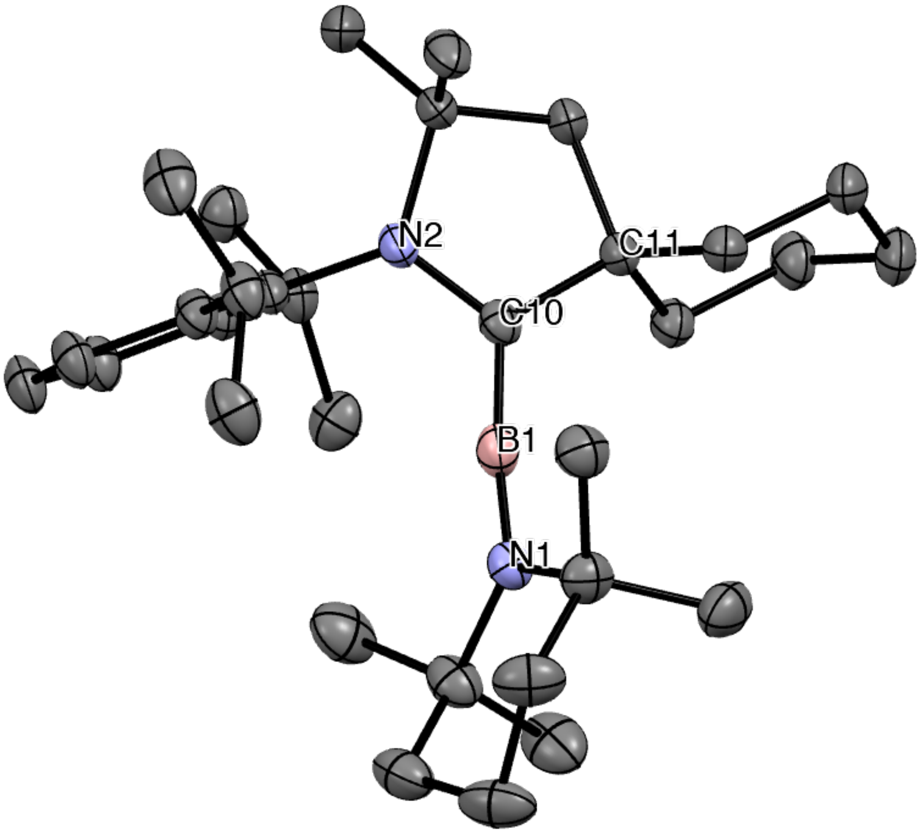

Supplementary Fig. 2. Crystal structure of [**3**]$^{+\bullet}$. Hydrogen atoms and counter anion are omitted for clarity. Thermal ellipsoids are set at 50% probability. Selected bond distances [Å], bond angles [°]: N2-C10 1.351(2), C11-C10 1.532(2), C10-B1 1.457(3), B1-N1 1.347(3), C10-B1-N1 172.56(19), N2-C10-C11 110.45(14), N2-C10-B1 124.67(16), C11-C10-B1 124.80(16).

Supplementary Table 2. Crystal data and experimental details for [**3**][$B(C_6F_5)_4$].

CCDC Number	2167149	
Empirical formula	C56 H53 B2 F20 N2	
Formula weight	1155.62	
Crystal system	Monoclinic	
Space group	*P2₁/c*	
Unit cell dimensions	a = 16.8135(9) Å	α= 90°.
	b = 20.2963(10) Å	β= 112.452(2)°.
	c = 16.4739(9) Å	γ = 90°.
Volume	5195.6(5) Å^3	
Z	4	
F(000)	2372	
Density (calculated)	1.477 Mg/m^3	
Wavelength	0.71073 Å	
Cell parameters reflections used	9477	
Theta range for Cell parameters	2.49 to 32.91°.	
Absorption coefficient	0.135 mm^{-1}	
Temperature	100(2) K	
Crystal size	0.300 x 0.200 x 0.030 mm^3	
Data collection and refinement		
Diffractometer	Bruker D8 VENTURE	
Absorption correction	Semi-empirical from equivalents	
Max. and min. transmission	0.9656 and 0.8983	
No. of measured reflections	102009	
No. of independent reflections	9138 [R(int) = 0.0595]	
No. of observed [I>2igma(I)]	8005	
Completeness to theta = 24.999°	100.0 %	
Theta range for data collection	1.672 to 24.999°.	
Final R indices [I>2sigma(I)]	R1 = 0.0378, wR2 = 0.0975	
R indices (all data)	R1 = 0.0438, wR2 = 0.1043	
Goodness-of-fit on F^2	1.044	
No. of reflections	9138	
No. of parameters	731	
No. of restraints	3	
Largest diff. peak and hole	0.565 and -0.262 e.Å^{-3}	

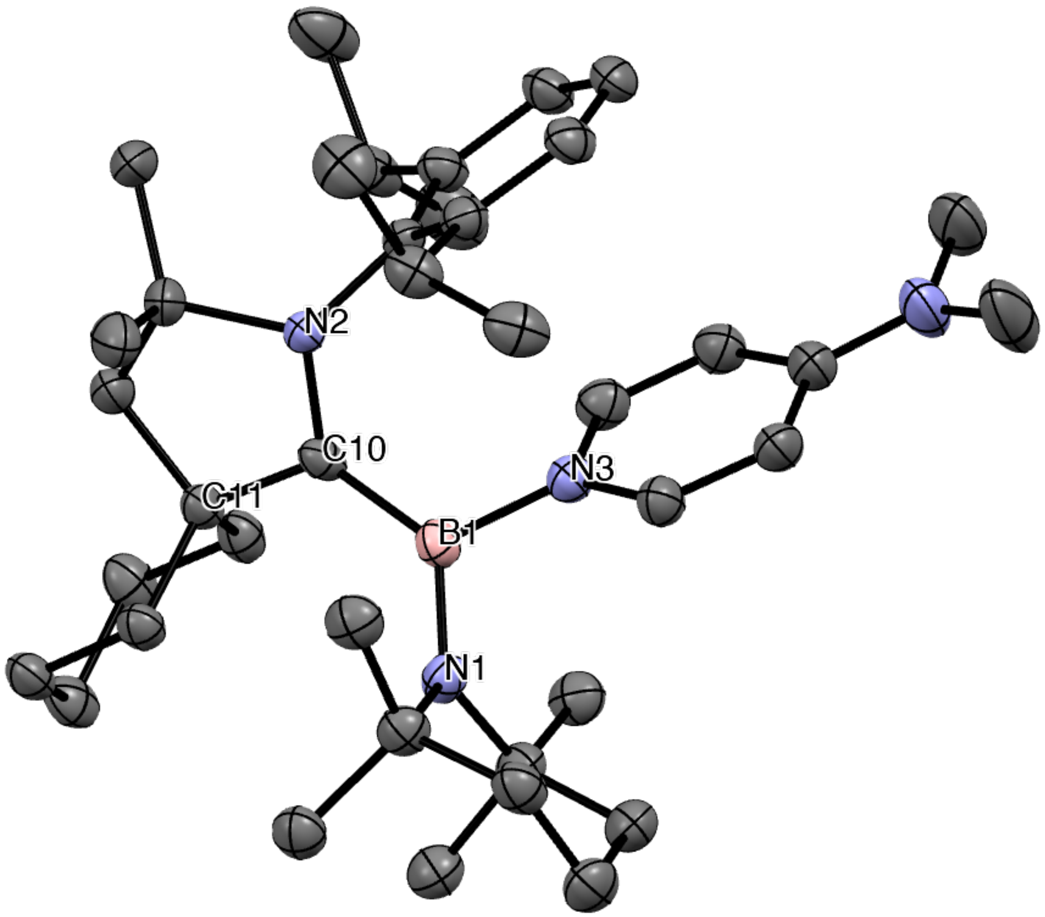

Supplementary Fig. 3. Crystal structure of [**6**]$^{+\bullet}$. Hydrogen atoms and counter anion are omitted for clarity. Thermal ellipsoids are set at 50% probability. Selected bond distances [Å], bond angles [°]:N2-C10 1.381(3), C10-C11 1.546(4), C10-B1 1.541(4), B1-N1 1.459(4), B1-N3 1.553(4), N2-C10-C11 106.8(2), N2-C10-B1 129.2(2), C11-C10-B1 123.9(2), C10-B1-N3 116.7(2), C10-B1-N1 129.8(3), N1-B1-N3 113.4(2).

Supplementary Table 3. Crystal data and structure refinement for [**6**][$B(C_6F_5)_4$].

CCDC Number	2167150	
Empirical formula	C63 H63 B2 F20 N4	
Formula weight	1277.79	
Crystal system	Triclinic	
Space group	*P-1*	
Unit cell dimensions	a = 13.2270(10) Å	α = 91.771(3)°.
	b = 14.0661(9) Å	β = 109.690(2)°.
	c = 16.9157(12) Å	γ = 94.635(2)°.
Volume	2947.8(4) $Å^3$	
Z	2	
F(000)	1318	
Density (calculated)	1.440 Mg/m^3	
Wavelength	0.71073 Å	
Cell parameters reflections used	8240	
Theta range for Cell parameters	2.30 to 28.00°.	
Absorption coefficient	0.128 mm^{-1}	
Temperature	100(2) K	
Crystal size	0.25 x 0.15 x 0.05 mm^3	
Data collection and refinement		
Diffractometer	Bruker D8 VENTURE	
Absorption correction	Semi-empirical from equivalents	
Max. and min. transmission	1.0000 and 0.8884	
No. of measured reflections	44141	
No. of independent reflections	10367 [R(int) = 0.0776]	
No. of observed [I>2igma(I)]	8145	
Completeness to theta = 24.998°	99.9 %	
Theta range for data collection	1.643 to 24.998°.	
Final R indices [I>2sigma(I)]	R1 = 0.0575, wR2 = 0.1529	
R indices (all data)	R1 = 0.0731, wR2 = 0.1690	
Goodness-of-fit on F^2	1.003	
No. of reflections	10367	
No. of parameters	805	
No. of restraints	0	
Largest diff. peak and hole	0.904 and -0.304 e.$Å^{-3}$	

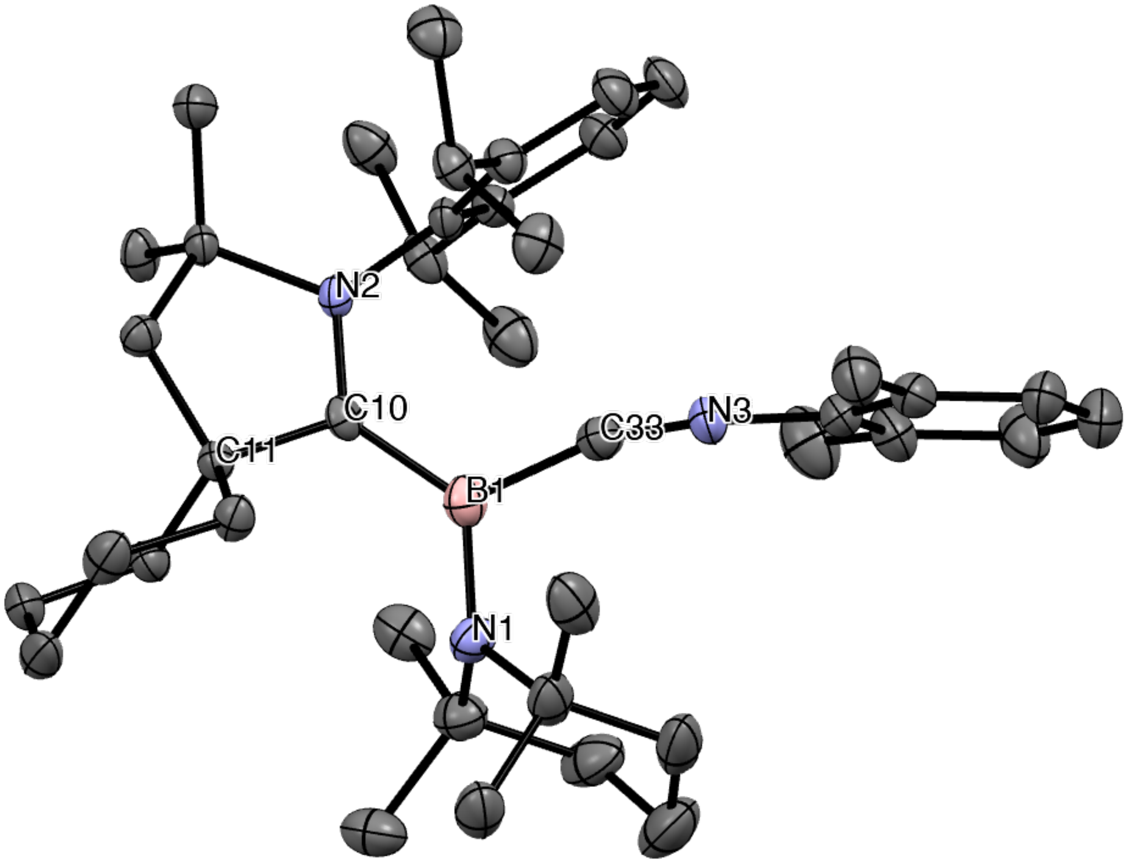

Supplementary Fig. 4. Crystal structure of [**9**]$^{+\bullet}$. Hydrogen atoms, solvents, and counter anion are omitted for clarity. Thermal ellipsoids are set at 50% probability. Selected bond distances [Å], bond angles [°]: C10-C11 1.533(4), N2-C10 1.332(3), C10-B1 1.558(4), B1-N1 1.464(4), B1-C33 1.588(5), C33-N3 1.159(4), C11-C10-N2 109.1(2), C11-C10-B1 125.0(2), N2-C10-B1 125.8(3), C10-B1-N1 127.0(3), C10-B1-C33 120.5(3), N1-B1-C33 112.4(3), B1-C33-N3 164.1(3).

Supplementary Table 4. Crystal data and structure refinement for [**9**][$B(C_6F_5)_4$].

CCDC Number	2167151	
Empirical formula	C65 H62 B2 F20 N3	
Formula weight	1286.79	
Crystal system	Triclinic	
Space group	*P-1*	
Unit cell dimensions	a = 11.7336(8) Å	α= 75.117(3)°.
	b = 14.9950(12) Å	β= 79.806(3)°.
	c = 19.2401(14) Å	γ = 89.256(3)°.
Volume	3218.1(4) $Å^3$	
Z	2	
F(000)	1326	
Density (calculated)	1.328 Mg/m^3	
Wavelength	0.71073 Å	
Cell parameters reflections used	9945	
Theta range for Cell parameters	2.23 to 27.25°.	
Absorption coefficient	0.117 mm^{-1}	
Temperature	100(2) K	
Crystal size	0.250 x 0.150 x 0.050 mm^3	
Data collection and refinement		
Diffractometer	Bruker D8 VENTURE	
Absorption correction	Semi-empirical from equivalents	
Max. and min. transmission	1.0000 and 0.8680	
No. of measured reflections	46867	
No. of independent reflections	11324 [R(int) = 0.0828]	
No. of observed [I>2igma(I)]	8170	
Completeness to theta = 24.999°	99.9 %	
Theta range for data collection	1.764 to 24.999°.	
Final R indices [I>2sigma(I)]	R1 = 0.0572, wR2 = 0.1219	
R indices (all data)	R1 = 0.0812, wR2 = 0.1357	
Goodness-of-fit on F^2	1.046	
No. of reflections	11324	
No. of parameters	823	
No. of restraints	0	
Largest diff. peak and hole	0.545 and -0.339 e.$Å^{-3}$	

1.4. NMR spectra

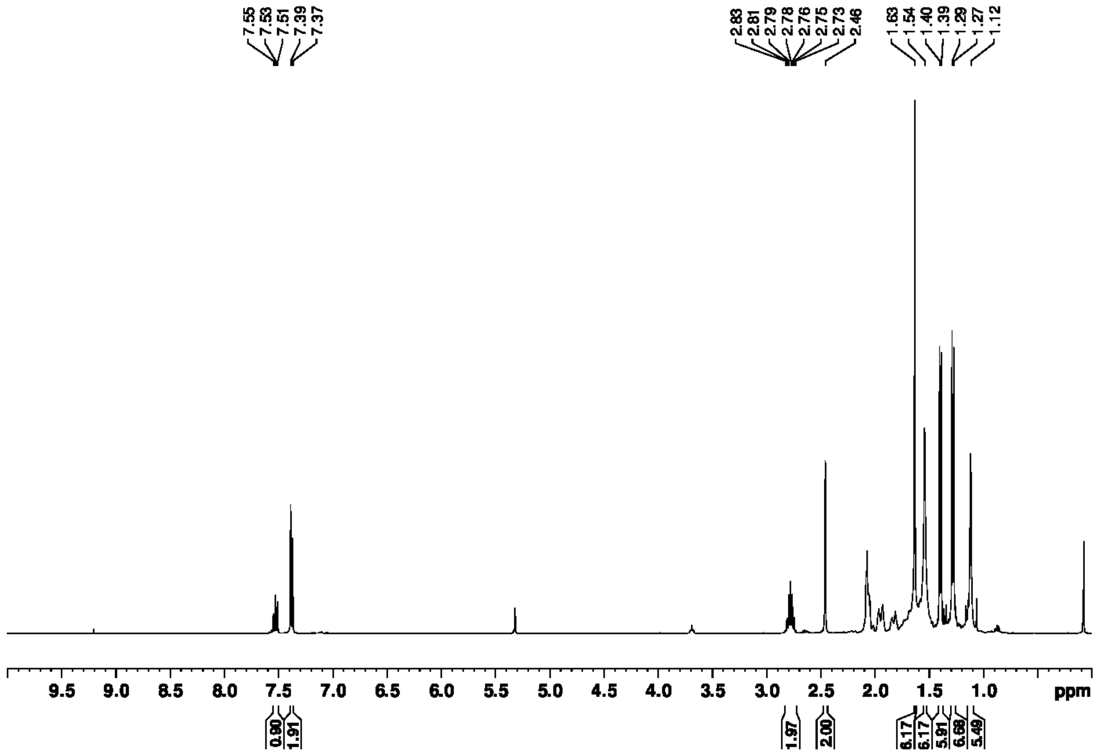

Supplementary Fig. 5. ^{1}H NMR spectrum of [**1**][OTf] in CD_2Cl_2.

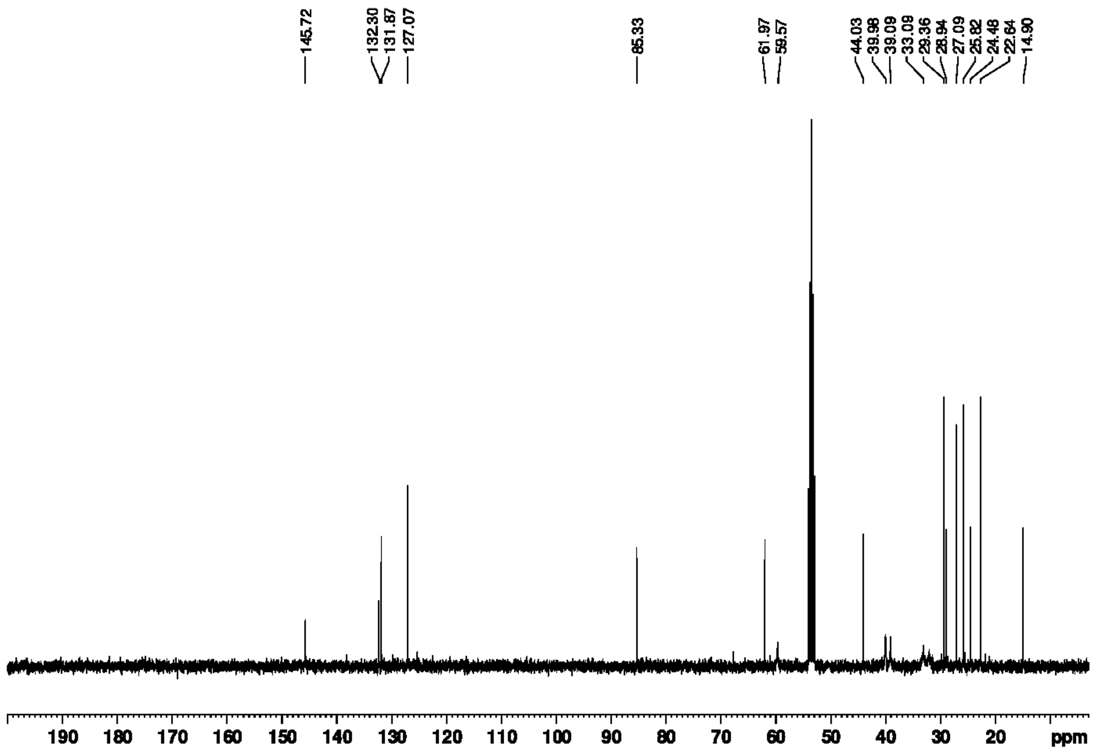

Supplementary Fig. 6. $^{13}C\{^{1}H\}$ NMR spectrum of [**1**][OTf] in CD_2Cl_2.

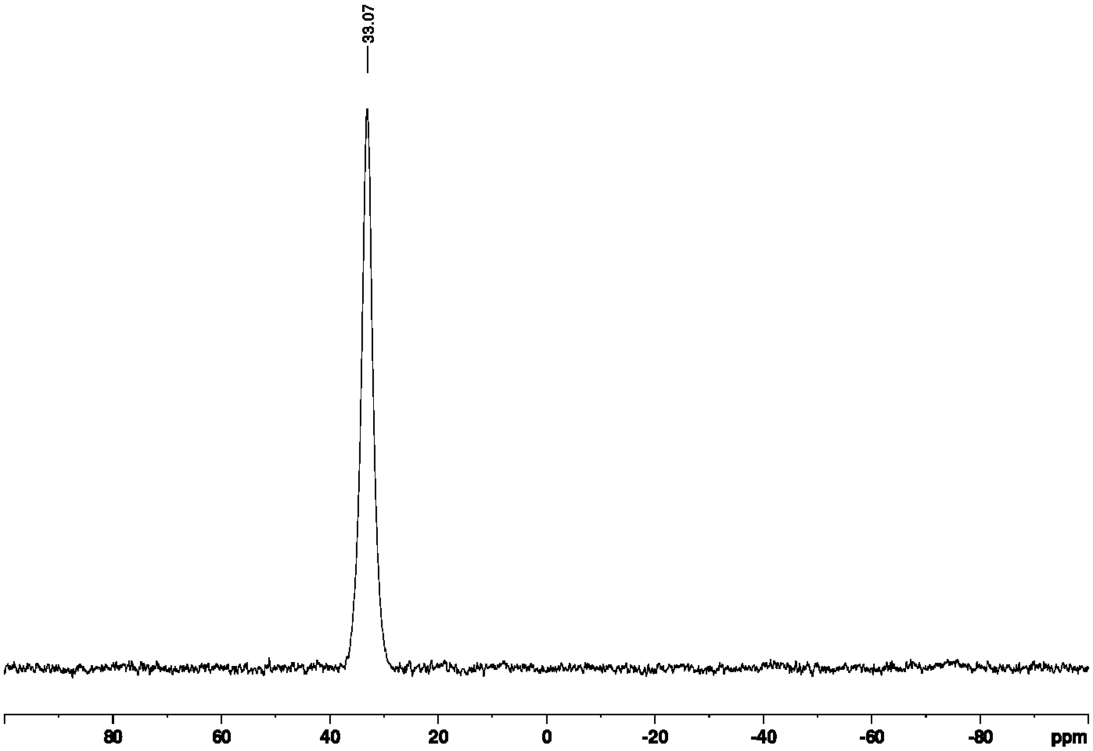

Supplementary Fig. 7. ^{11}B NMR spectrum of [**1**][OTf] in CD_2Cl_2.

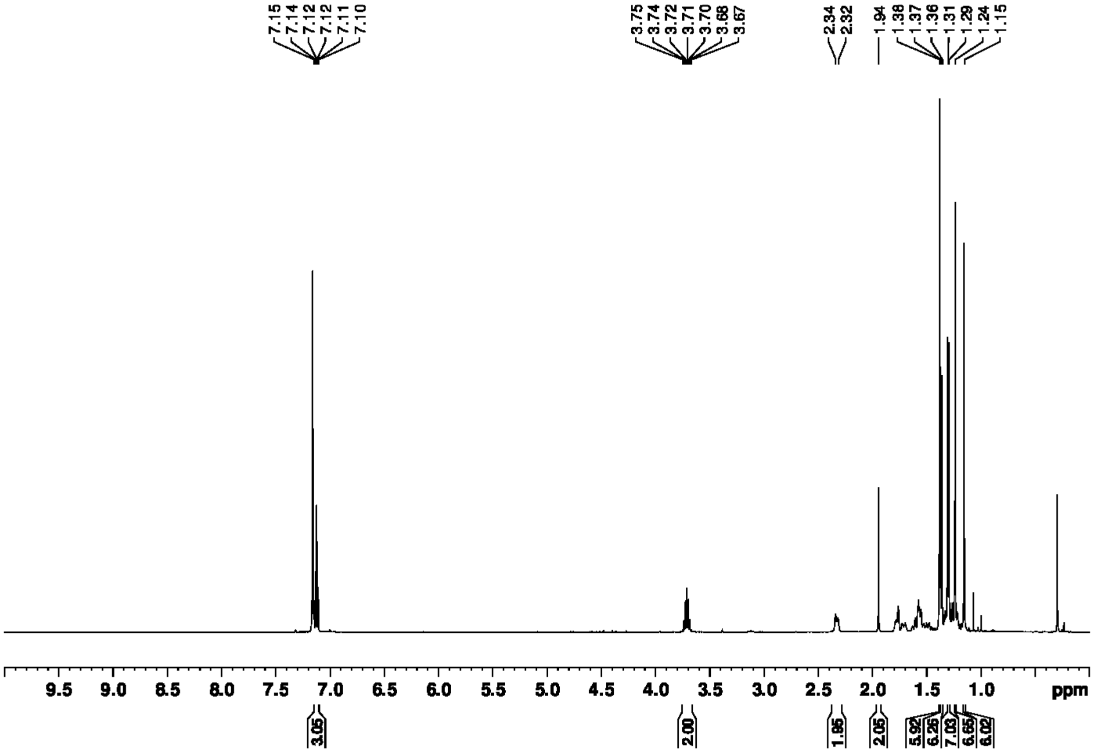

Supplementary Fig. 8. ^{1}H NMR spectrum of **4** in C_6D_6.

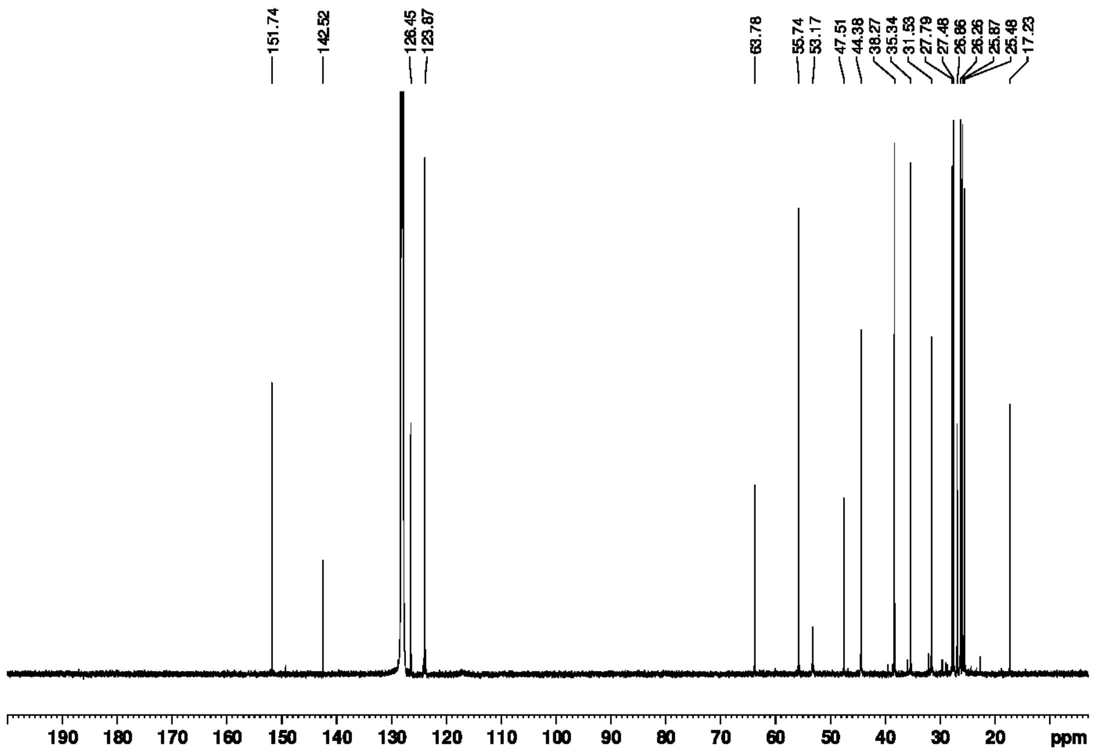

Supplementary Fig. 9. $^{13}C\{^{1}H\}$ NMR spectrum of **4** in C_6D_6.

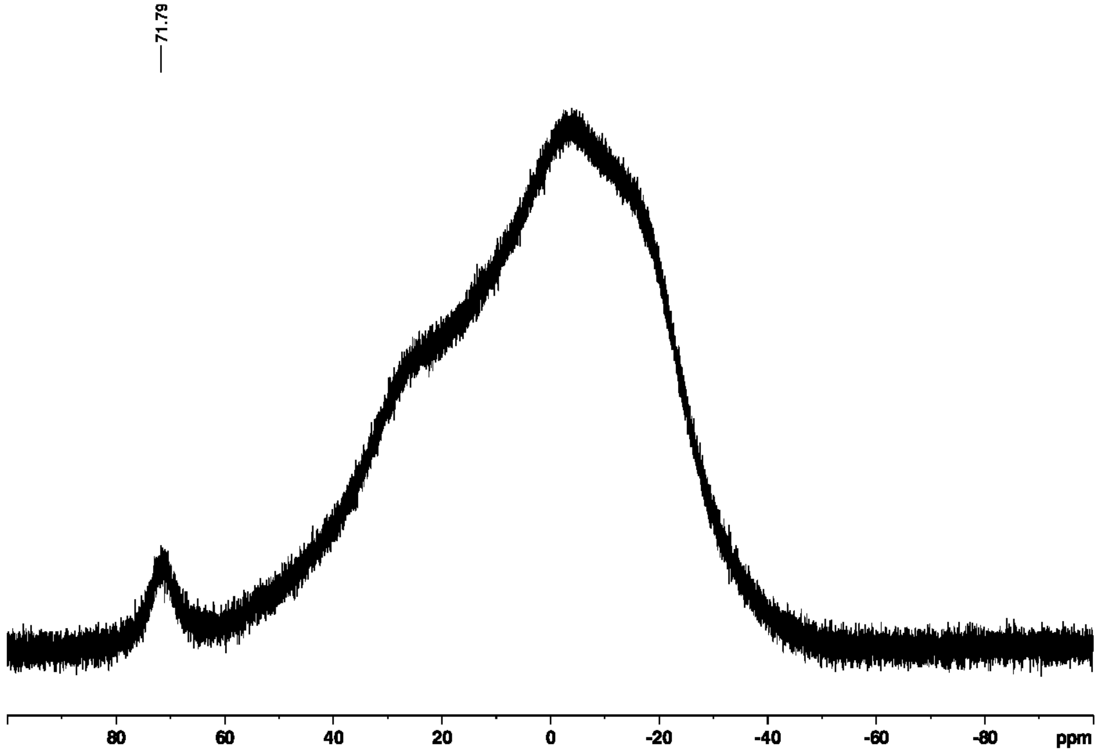

Supplementary Fig. 10. ^{11}B NMR spectrum of **4** in C_6D_6.

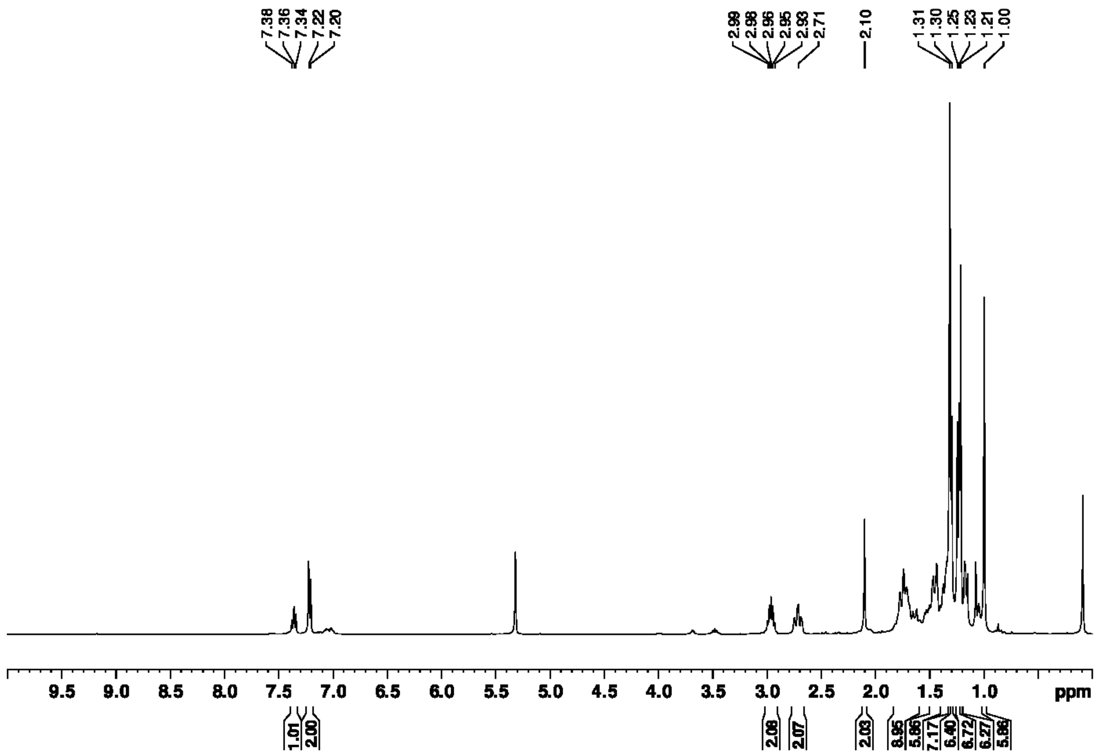

Supplementary Fig. 11. ^{1}H NMR spectrum of **7** in CD_2Cl_2.

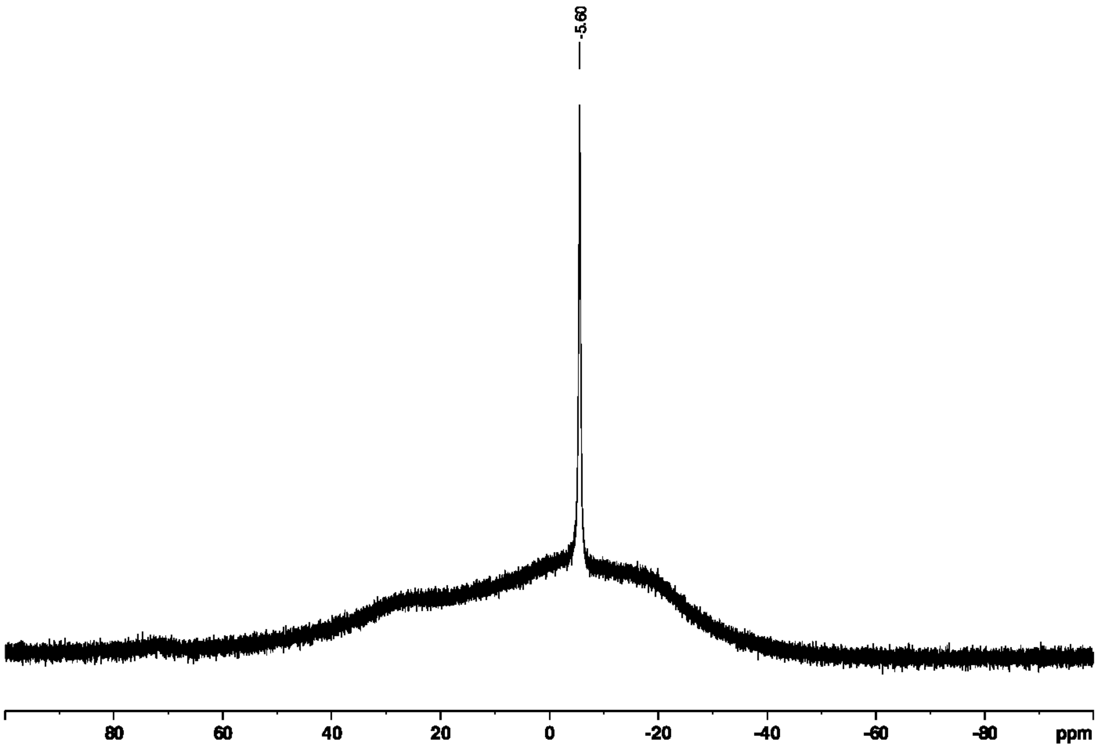

Supplementary Fig. 12. ^{11}B NMR spectrum of **7** in CD_2Cl_2.

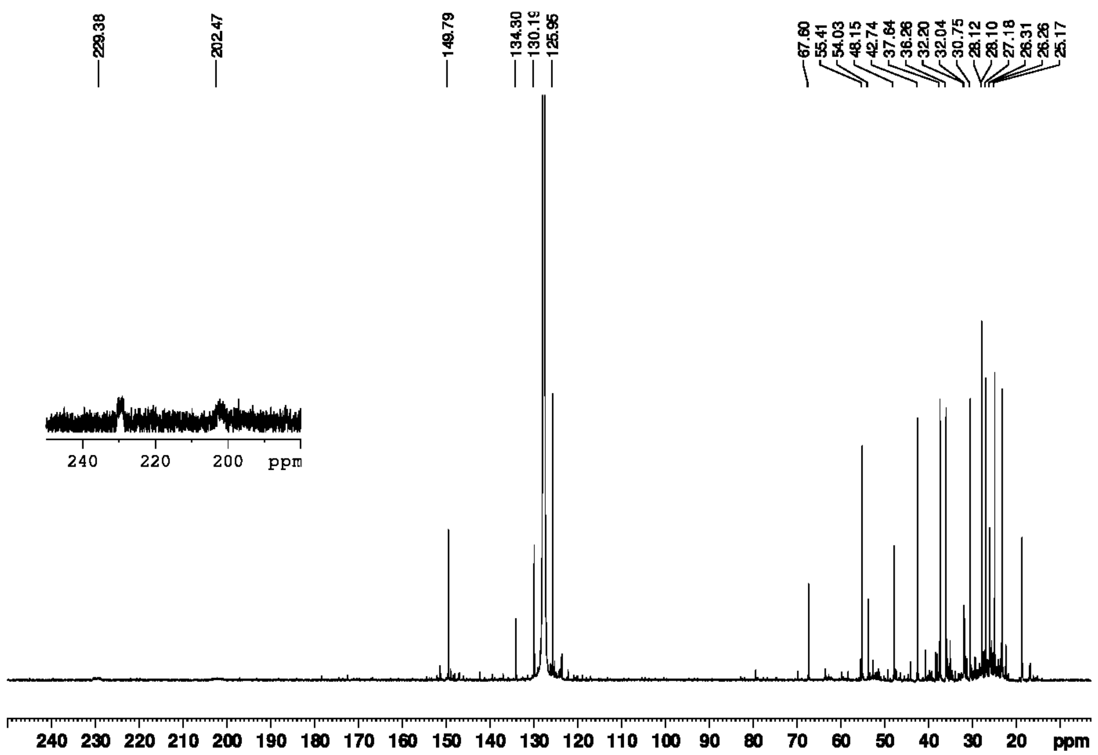

Supplementary Fig. 13. $^{13}C\{^{1}H\}$ NMR spectrum of **7** in C_6D_6.

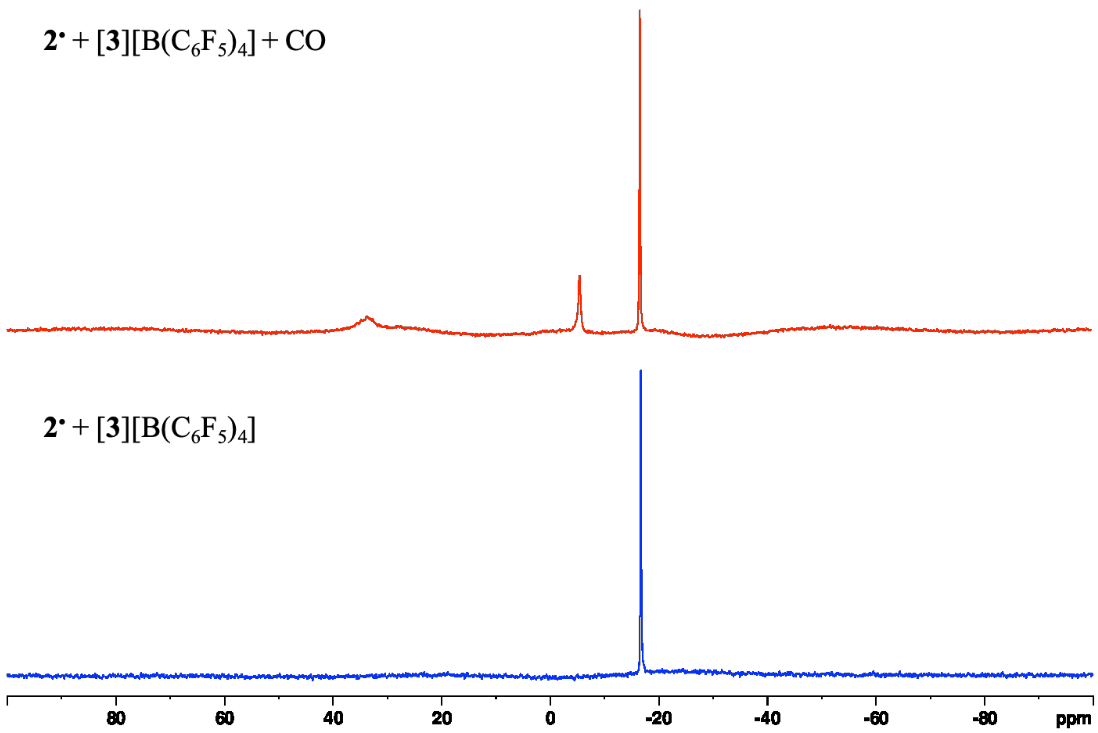

Supplementary Fig. 14. ^{11}B NMR spectrum of an equimolar mixture of **2**• and [**3**][$B(C_6F_5)_4$] in CD_2Cl_2 before and after CO exposure.

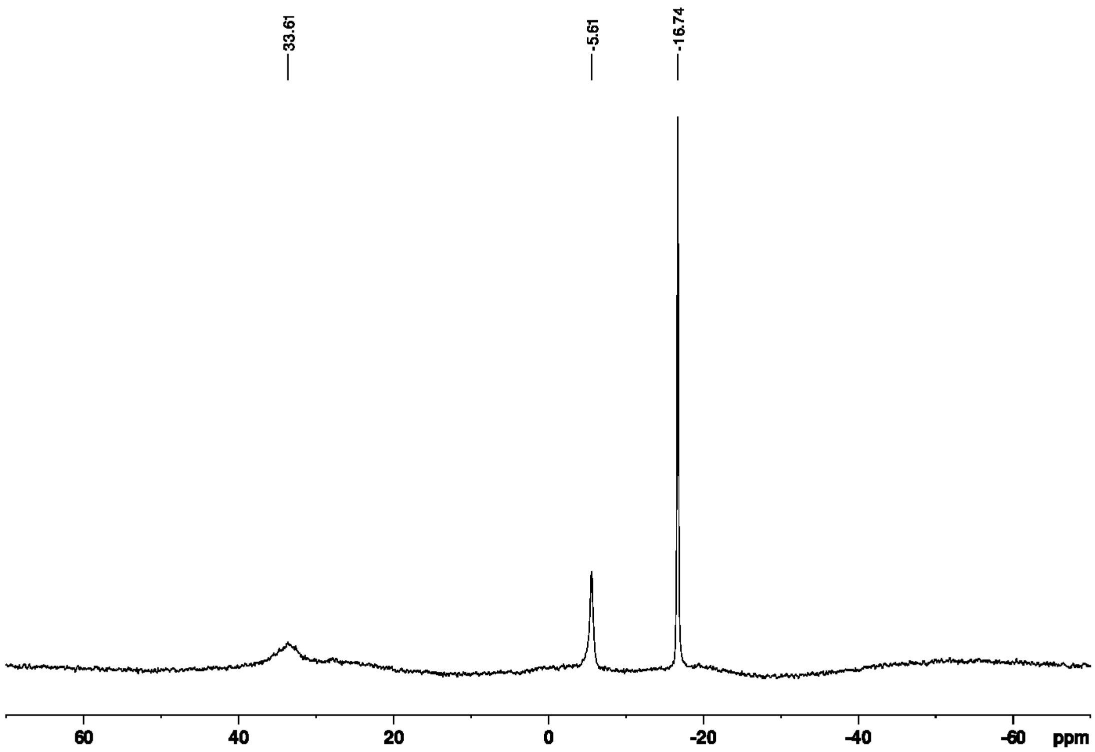

Supplementary Fig. 15. ^{11}B NMR spectrum (in CD_2Cl_2) of [**1**][$B(C_6F_5)_4$] and **7** obtained from the reaction of **2**• and [**3**][$B(C_6F_5)_4$] with CO.

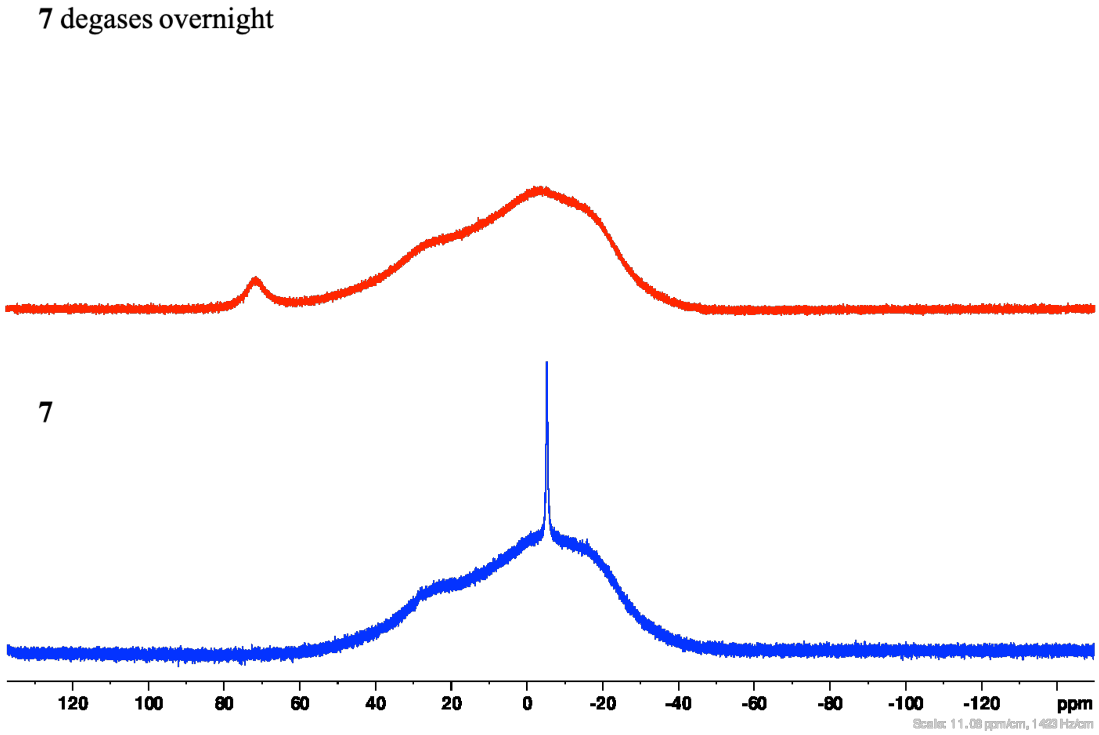

Supplementary Fig. 16. ^{11}B NMR spectrum (in C_6D_6) of **4** obtained from the removal of CO from **7** under vacuum.

1.5. Electrochemical Measurement

All electrochemical experiments were performed with an electrochemical analyzer from CH Instruments (Model 660 D) at a scan rate of 100 mV/s with a standard 3-electrode system: an $Ag/AgNO_3$ reference electrode, a glassy carbon working electrode, and a platinum wire auxiliary electrode. Electrode was immersed in a solution (10 mL) containing $^nBu_4NPF_6$ (0.1 M) and analyte (0.001 M in CH_2Cl_2). The supporting electrolyte, *n*-Bu_4NPF_6, was dried at 80 °C under vacuum for overnight, and was stored inside a glove box. In all cases, ferrocene was used as an internal standard, and all reduction potentials are reported with respect to the $E_{1/2}$ of the Fc/Fc^+ redox couple.

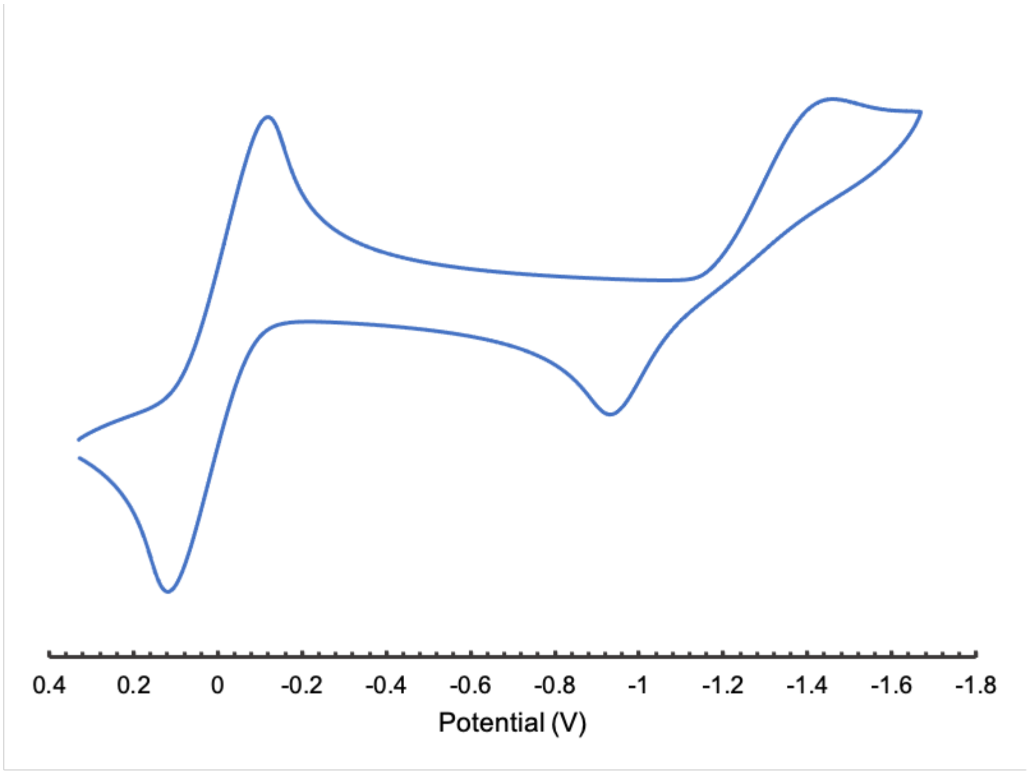

Supplementary Fig. 17. Cyclic voltammogram of [**1**][OTf] with ferrocene as internal reference.

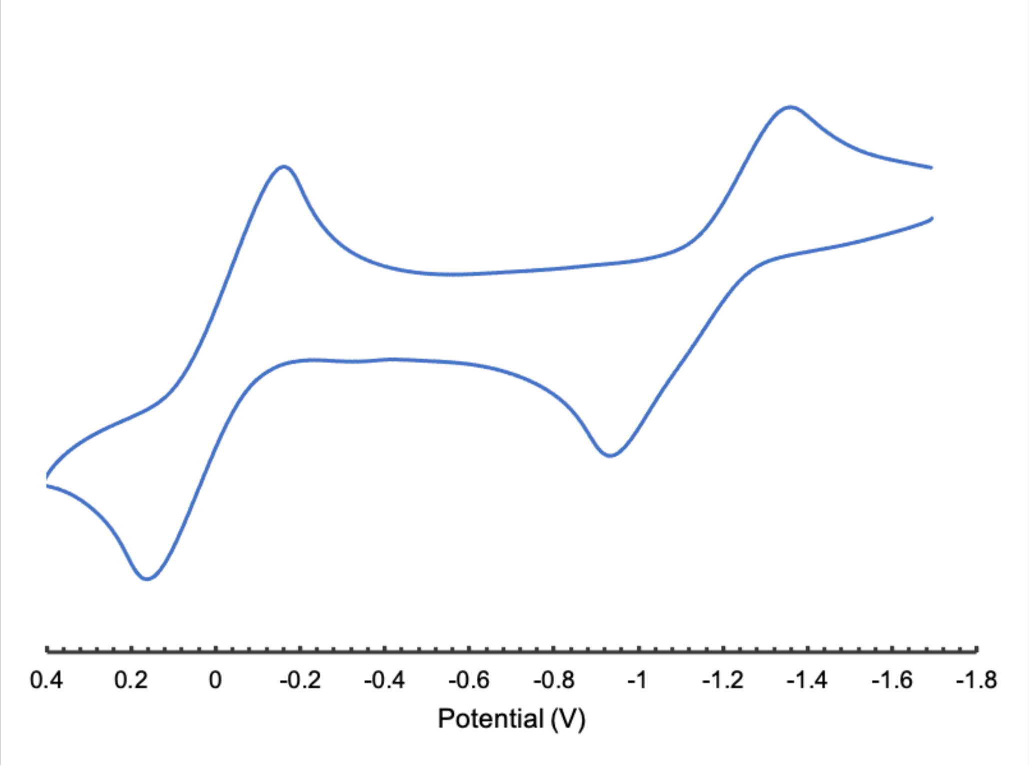

Supplementary Fig. 18. Cyclic voltammogram of **2**$^{\bullet}$ with ferrocene as internal reference.

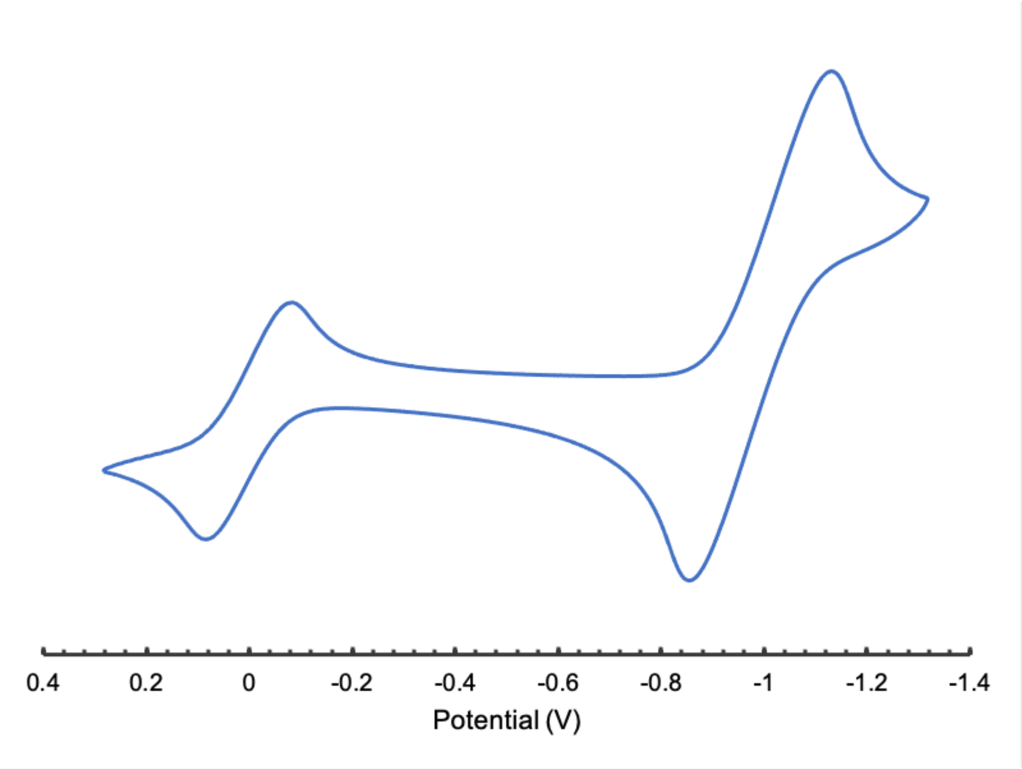

Supplementary Fig. 19. Cyclic voltammogram of [**3**][B(C_6F_5)$_4$] with ferrocene as internal reference.

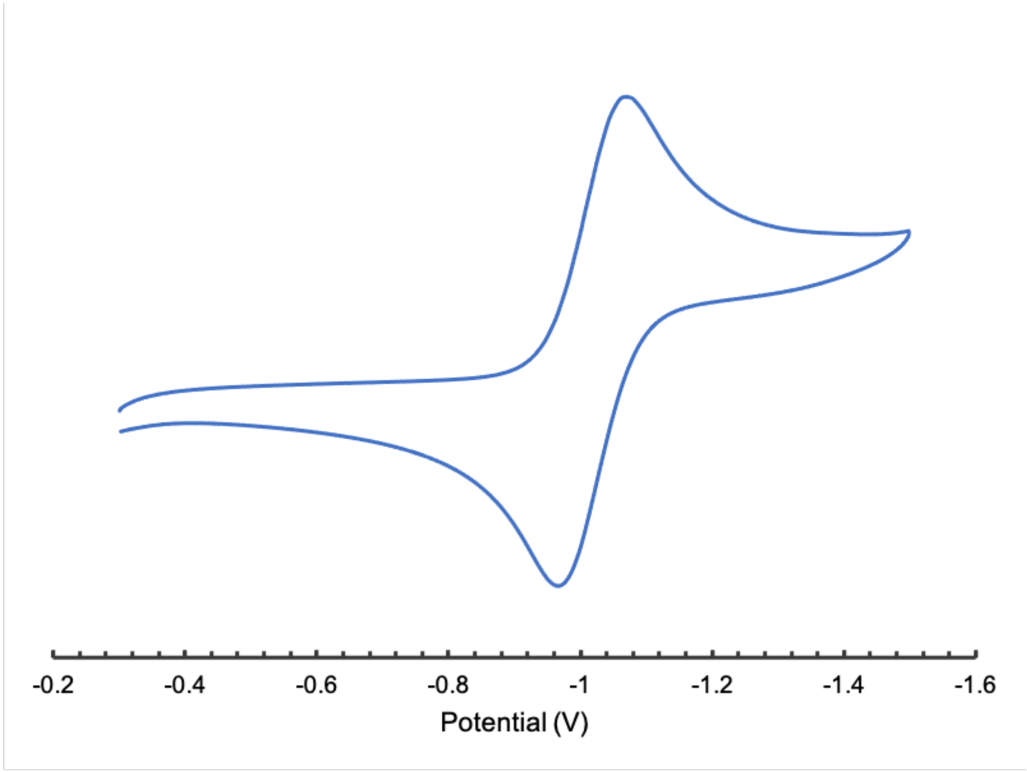

Supplementary Fig. 20. Cyclic voltammogram of [**9**][B(C_6F_5)$_4$] vs. ferrocene/ferrocenium.

1.6. EPR Spectra and Simulations

Room temperature solution EPR measurement was carried out on a Bruker EMXnano spectrometer. Samples containing around 0.002 M of radical species were transferred to a quartz J-Young's tube under nitrogen atmosphere. EPR spectra of **2**$^{\bullet}$, [**3**][$B(C_6F_5)_4$], and [**9**][$B(C_6F_5)_4$] were obtained with a microwave power of 0.3162 mW, modulation amplitude of 1 G, time constant of 1.28 ms, and sweep time of 10 s. Low temperature EPR measurement of [**6**][$B(C_6F_5)_4$] was carried out on a Bruker EMXmicro spectrometer at 77K. A solution containing around 0.001M of [**6**][$B(C_6F_5)_4$] was transferred to a quartz J-Young's tube under nitrogen atmosphere. EPR spectrum of [**6**][$B(C_6F_5)_4$] was obtained with a microwave power of 21.10 mW, modulation amplitude of 0.25 G, time constant of 0.01 ms, and sweep time of 20 s. All reported EPR spectra were simulated using Easyspin5.2.30[4] with hyperfine coupling constants listed in the figure caption.

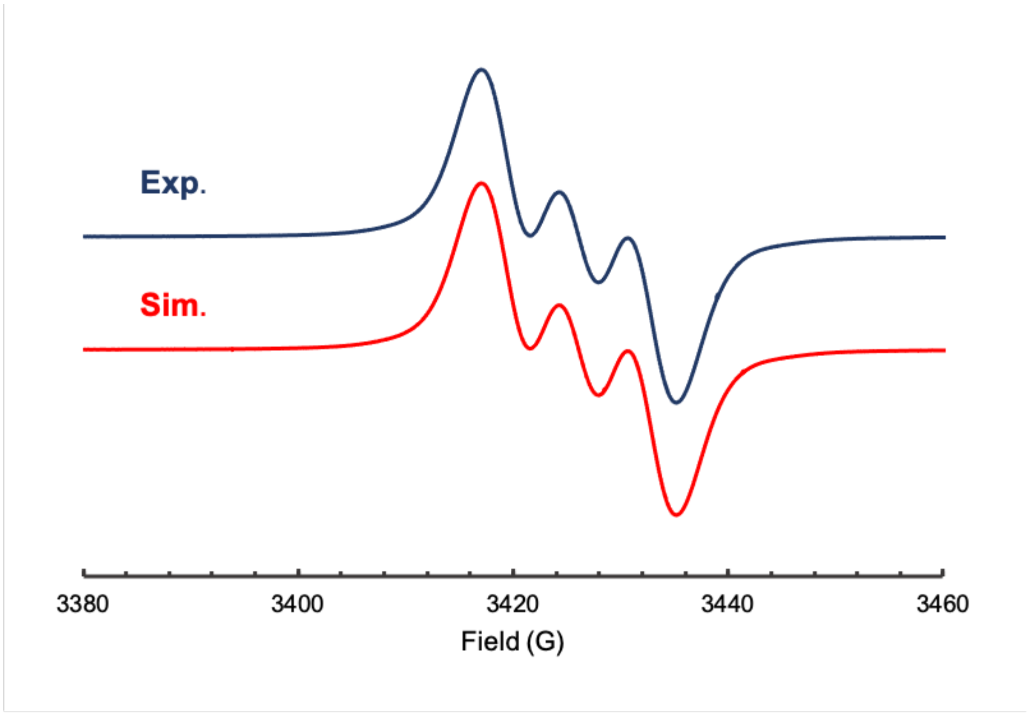

Supplementary Fig. 21. EPR spectrum of **2**$^{\bullet}$ in DCM at room temperature. The simulation parameters are g_{iso} = 2.0047, $a(^{14}N_{CAAC})$ = 6.62 G, and $a(^{11,10}B)$ = 0.77/0.26 G.

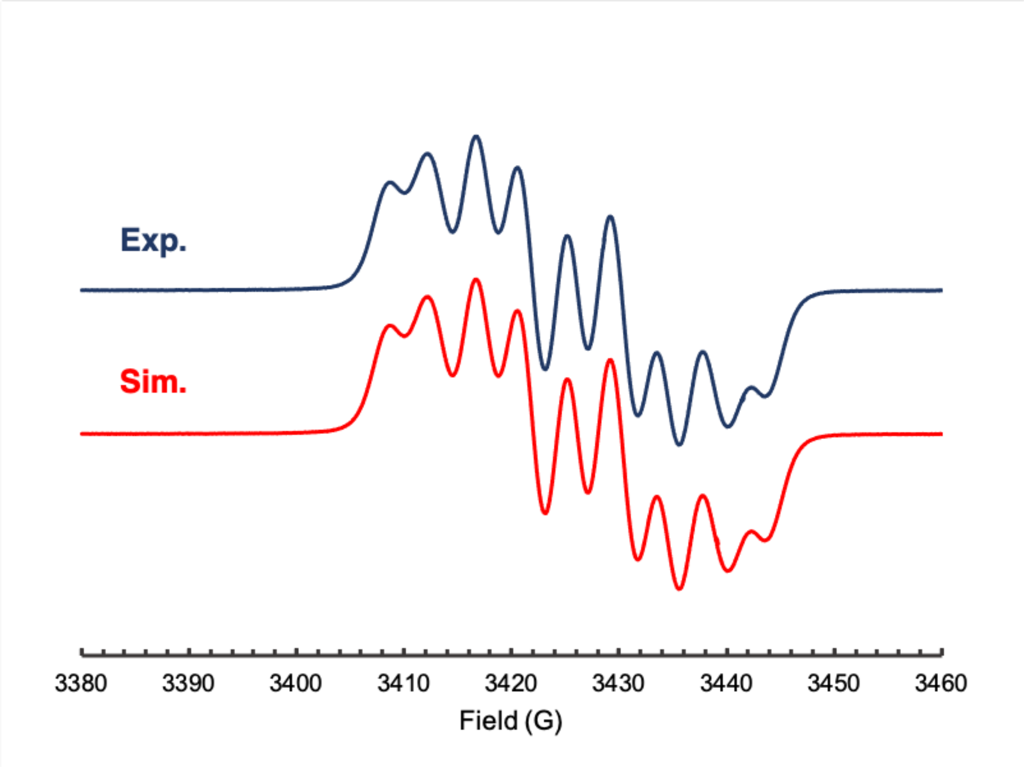

Supplementary Fig. 22. EPR spectrum of [**3**][B(C_6F_5)$_4$] in DCM at room temperature. The simulation parameters are g_{iso} = 2.0047, a(^{11,10}B) = 3.82/1.27 G, a($^{14}N_{CAAC}$) = 8.32 G, and a($^{14}N_{TMP}$) = 2.42 G.

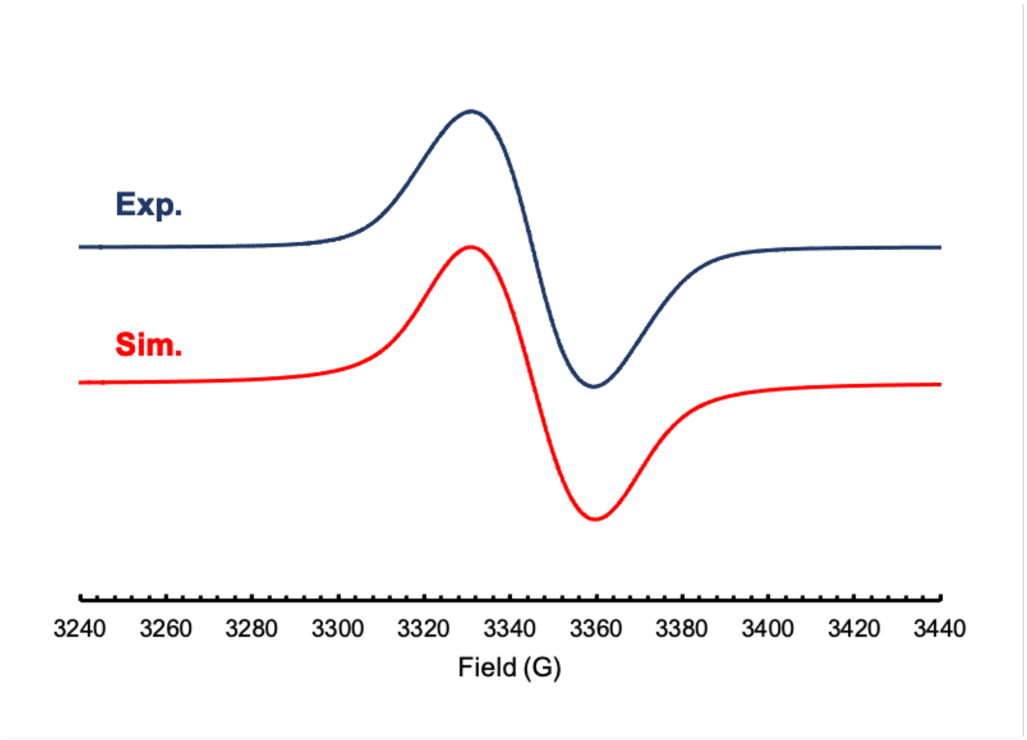

Supplementary Fig. 23. EPR spectrum of [**6**][B(C_6F_5)$_4$] in DCM at 77 K. The simulation parameters are g_{iso} = 2.0029, a(^{11,10}B) = 5.33/1.79 G, a($^{14}N_{cAAC}$) = 8.29 G, a($^{14}N_{TMP}$) = 2.63 G, and a($^{14}N_{DMAP}$) = 1.96 G.

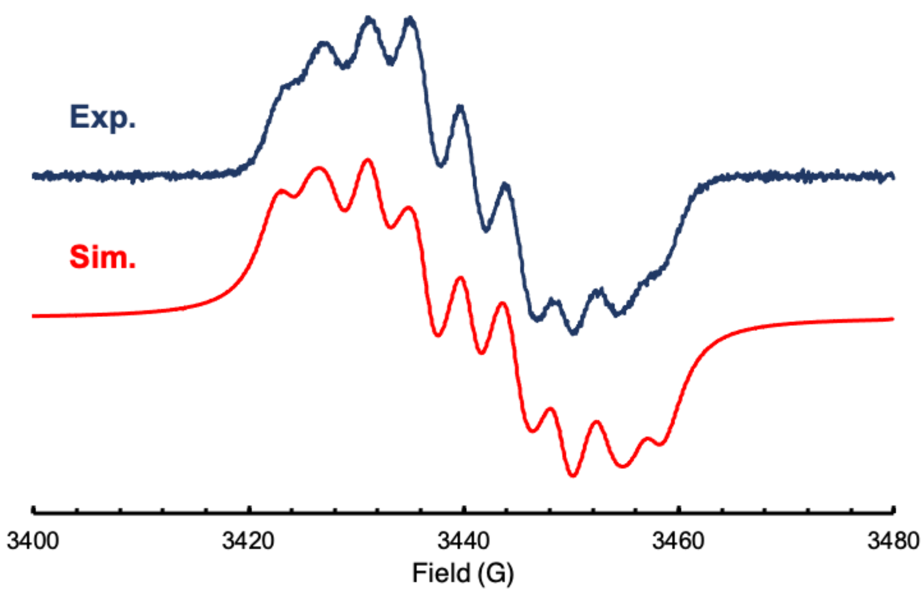

Supplementary Fig. 24. EPR spectrum of [**9**][B(C_6F_5)$_4$] in DCM at room temperature. The simulation parameters are g_{iso} = 2.0032, a(^{11,10}B) = 4.09/1.36 G, a($^{14}N_{cAAC}$) = 8.38 G, and a($^{14}N_{CNXyl}$) = 2.39 G.

1.7. Computational Details

Density functional theory (DFT) calculations (full geometry optimization) were carried out with Gaussian 16 program package[5] at the theory level of UCAM-B3LYP/6-31G(d,p)/SMD(CH_2Cl_2). Frequency calculations were performed to verify the optimized structures as minima. The molecular orbital surface was generated by GaussView 6.[6] The redox potentials are calculated in terms of Gibbs free energy change of the corresponding solvated redox reaction.[7] The hydride-ion affinity (HIA) was carried out with Gaussian 16 program package at the theory level of UM06-2X/6-311G(d,p)/PCM(CH_2Cl_2) in order to maintain consistency with the reported article by Ingleson's group.[8]

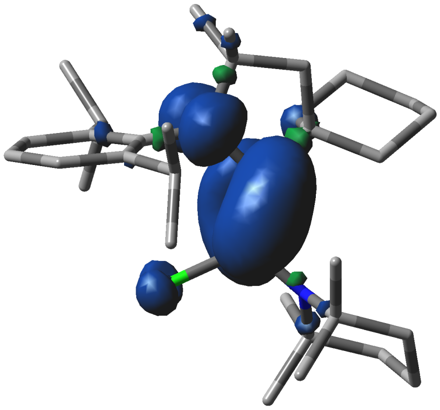

$\mathbf{2}^{\bullet}$

N_{cAAC}: 19.8%, C_{cAAC}: 47.6%, B: 31.7%, Cl: 0.1%, N_{TMP}: 0.1%

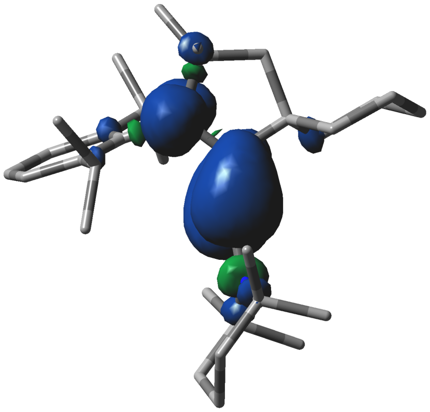

$[\mathbf{3}]^{+\bullet}$

N_{cAAC}: 33.6%, C_{cAAC}: 18.8%, B: 46.4%, N_{TMP}: 0.3%

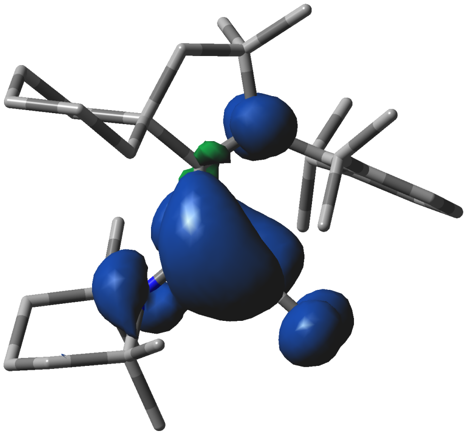

$[\mathbf{8}]^{+\bullet}$

N_{cAAC}: 5.5%, C_{cAAC}: 0.2%, B: 67.8%, C_{CO}: 14.5%, O_{CO}: 9.8%, N_{TMP}: 2.1%

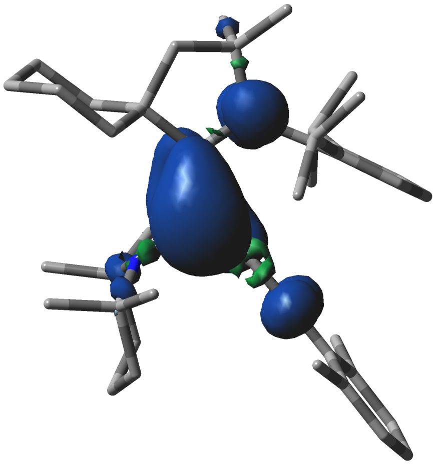

$[\mathbf{9}]^{+\bullet}$

N_{cAAC}: 13.2%, C_{cAAC}: 2.0%, B: 79.9%, C_{CNXyl}: 0.0%, N_{CNXyl}: 4.5%, N_{TMP}: 0.1%

Supplementary Fig. 25. Spin density plots of $\mathbf{2}^{\bullet}$, $[\mathbf{3}]^{+\bullet}$, $[\mathbf{8}]^{+\bullet}$, and $[\mathbf{9}]^{+\bullet}$ (isovalue = 0.002). Hydrogen atoms are omitted for clarity.

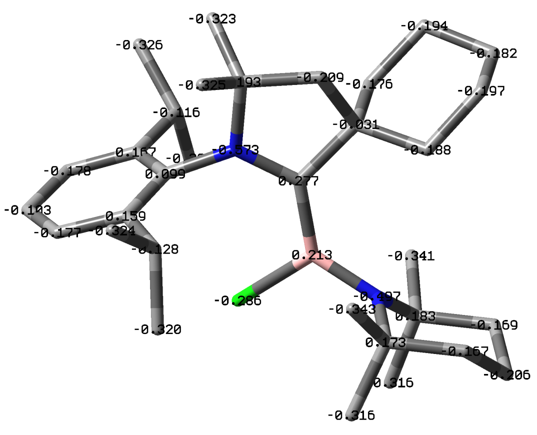

2$^{\bullet}$

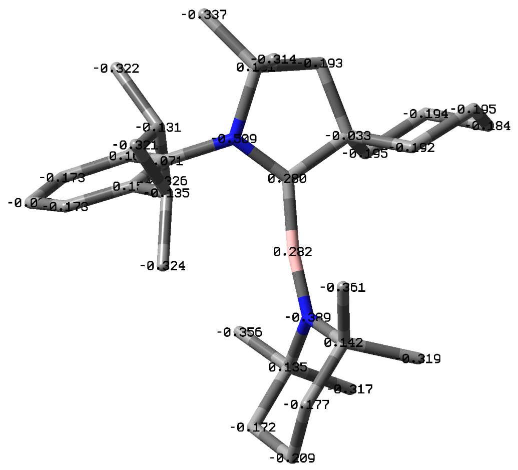

[**3**]$^{+\bullet}$

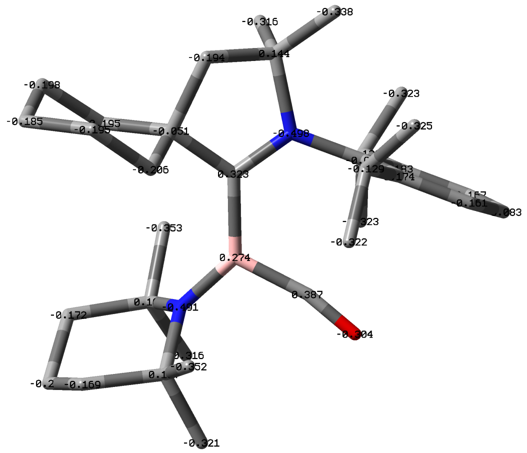

[**8**]$^{+\bullet}$

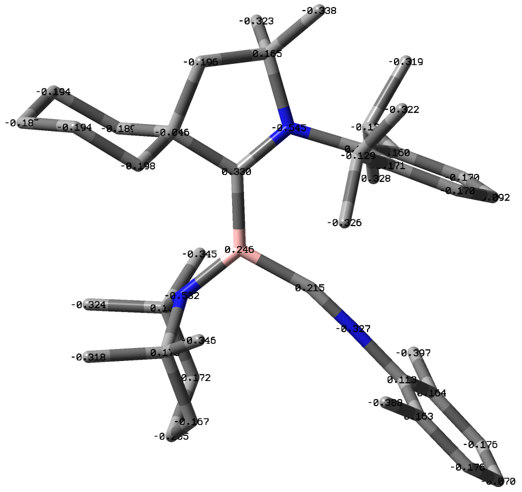

[**9**]$^{+\bullet}$

Supplementary Fig. 26. Mulliken charges of **2**$^{\bullet}$, [**3**]$^{+\bullet}$, [**8**]$^{+\bullet}$, and [**9**]$^{+\bullet}$. Hydrogen atoms are omitted for clarity.

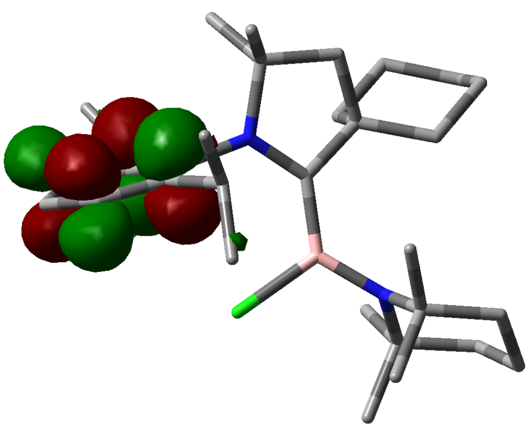

LUMO+1 (1.46)

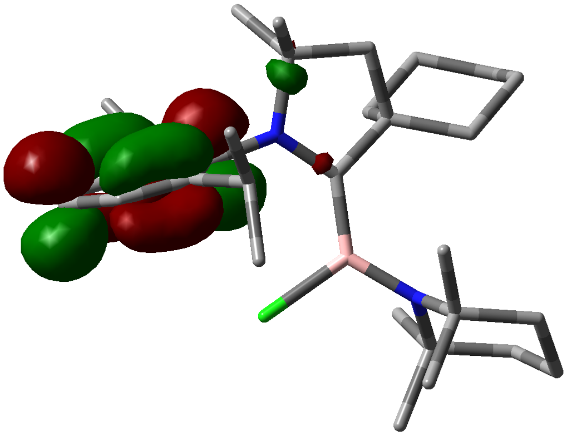

LUMO (1.35)

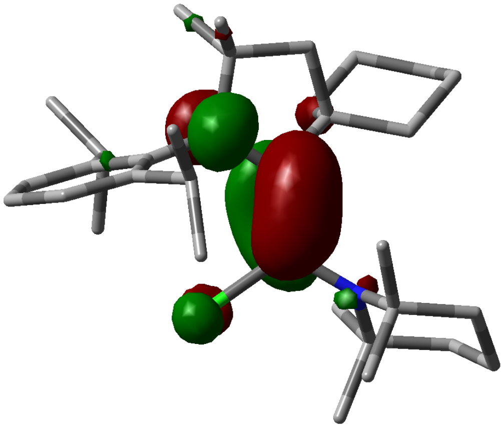

SOMO (-5.56)

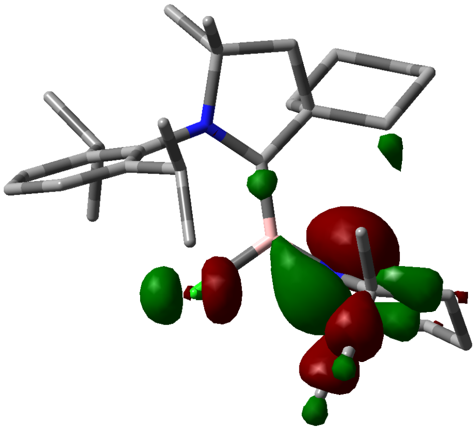

SOMO-1 (-6.72)

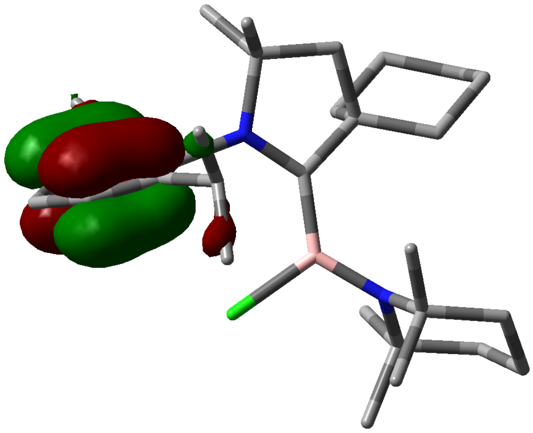

SOMO-2 (-7.64)

Supplementary Fig. 27. Selected molecular orbitals of **2**$^{\bullet}$ (isovalue = 0.05). Hydrogen atoms are omitted for clarity.

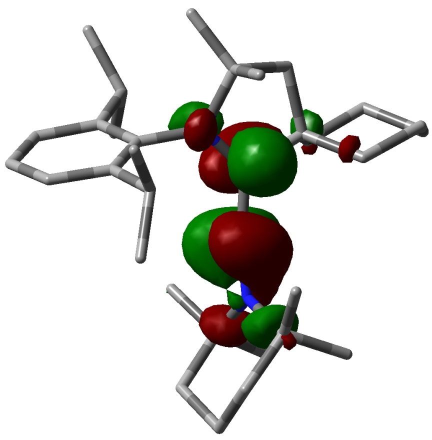

LUMO+3 (1.50)

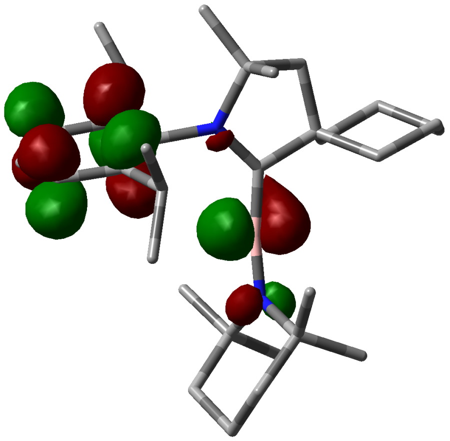

LUMO+2 (1.02)

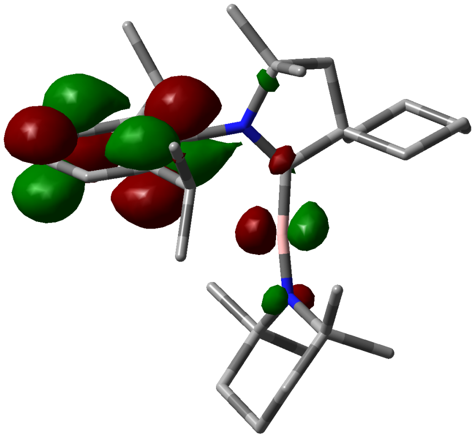

LUMO+1 (0.87)

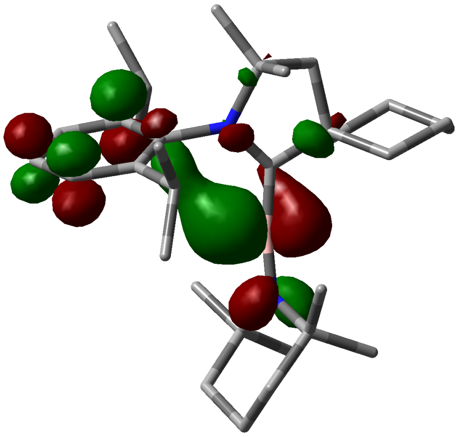

LUMO (0.66)

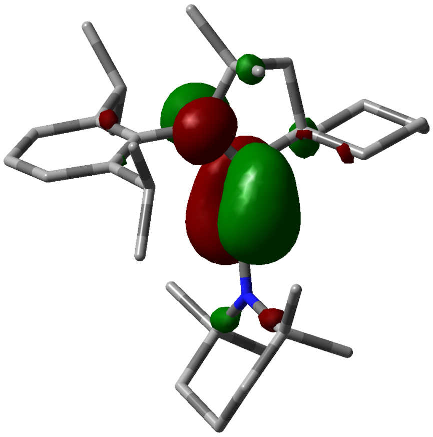

SOMO (-7.01)

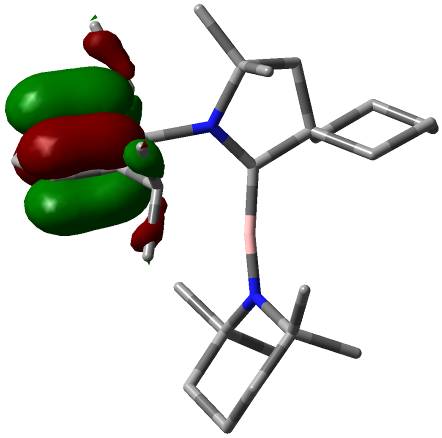

SOMO-1 (-8.15)

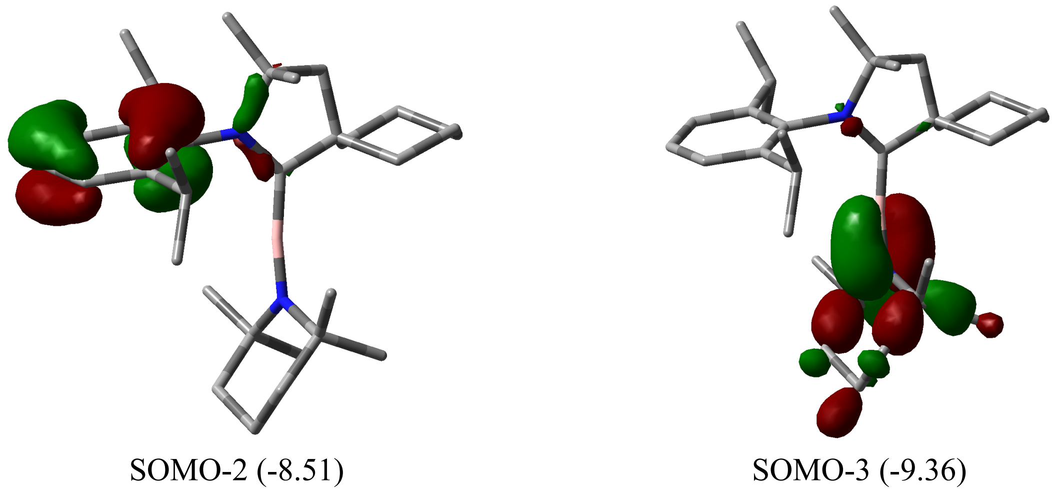

Supplementary Fig. 28. Selected molecular orbitals of [**3**]$^{+\bullet}$ (isovalue = 0.05). Hydrogen atoms are omitted for clarity.

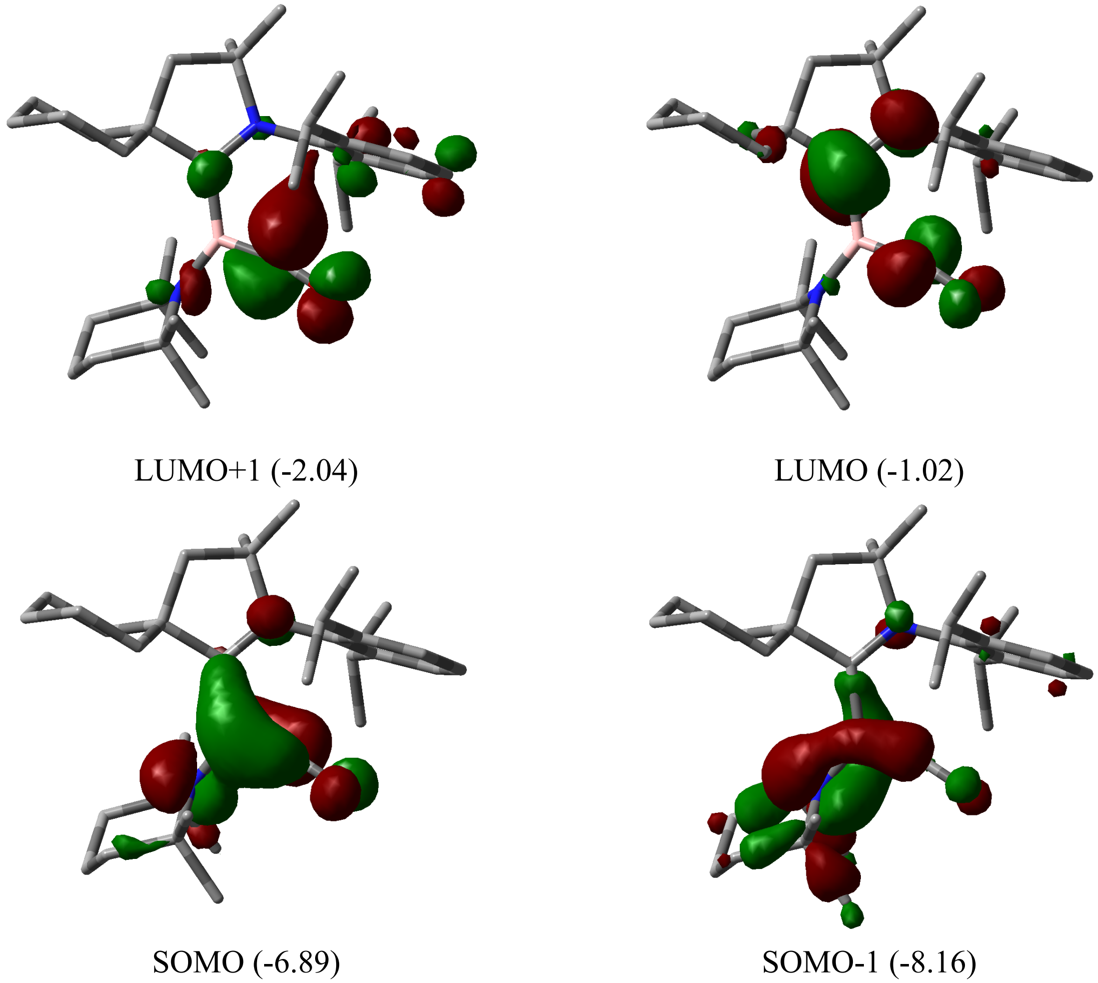

Supplementary Fig. 29. Selected molecular orbitals of [**8**]$^{+\bullet}$ (isovalue = 0.05). Hydrogen atoms are omitted for clarity.

Supplementary Table 5. Calculated Gibbs free energy (Hartree) at UCAM-B3LYP/6-31G**/SMD(CH_2Cl_2) level.

	Gibbs free energy (Hartree)
[**1**]$^{+}$	-1844.324352
**2**$^{\bullet}$	-1844.460150
[**3**]$^{+\bullet}$	-1384.088845
**4**	-1384.217597
[**8**]$^{+\bullet}$	-1497.345888
**7**	-1497.495492
[**9**]$^{+\bullet}$	-1786.840474
**10**	-1786.967953

Supplementary Table 6. Calculated redox potential (eV) at UCAM-B3LYP/6-31G**/SMD(CH_2Cl_2) level.

Reaction	$\Delta G°$	$E_{1/2}$ (vs. SHE^{a})	$E_{1/2}$ (vs. Fc/Fc^{+b})
[**1**]$^{+}$ + e^{-} → **2**$^{\bullet}$	-3.69525	-0.58	-0.98
[**3**]$^{+\bullet}$ + e^{-} → **4**	-3.50352	-0.78	-1.18
[**8**]$^{+\bullet}$ + e^{-} → **7**	-4.07093	-0.21	-0.61
[**9**]$^{+\bullet}$ + e^{-} → **10**	-3.46888	-0.81	-1.21

a. $\Delta G°_{SHE}$ = -4.28 eV.[9] *b*. Fc/Fc^{+}, $E_{1/2}$ = 0.40 V (vs. SHE).

Supplementary Table 7. Relative hydride-ion affinity of [**3**]$^{+\bullet}$ at UM06-2X/6-311G**/PCM(CH2Cl2) level.

Boron center Lewis acid	HIA (Kcal mol^{-1})
BEt_3	0.0
[Mes_2B]$^{+}$	-63.6
[**3**]$^{+\bullet}$	-29.4

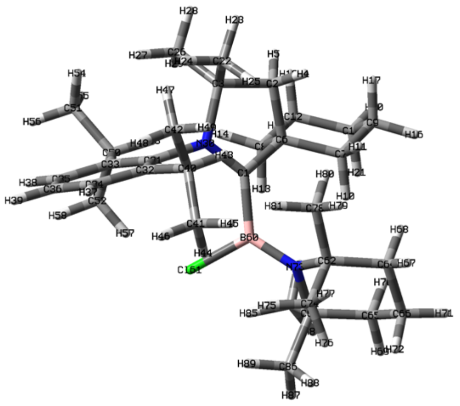

[**1**]$^{+}$

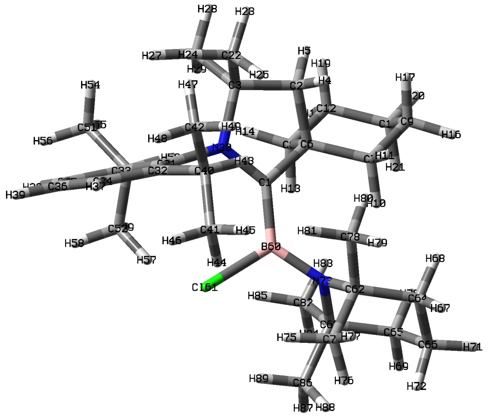

2$^{\bullet}$

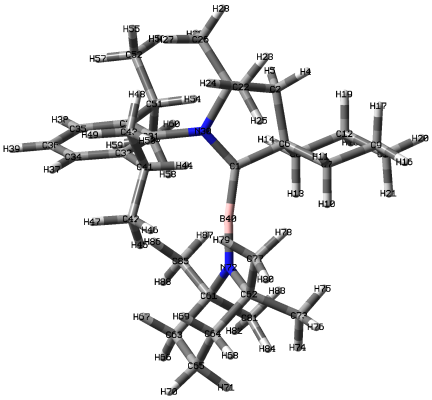

[**3**]$^{+\bullet}$

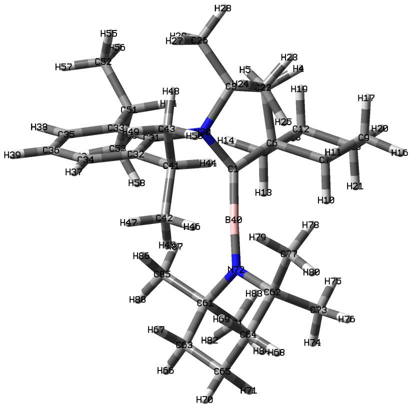

4

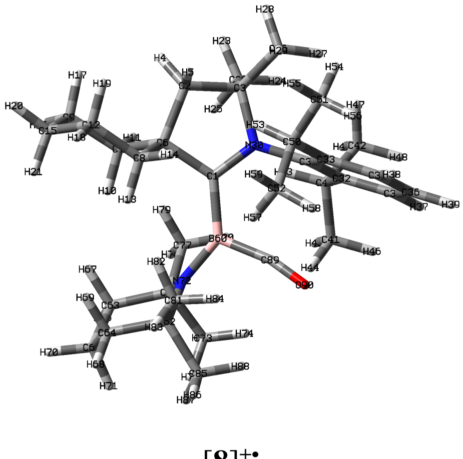

[**8**]$^{+\bullet}$

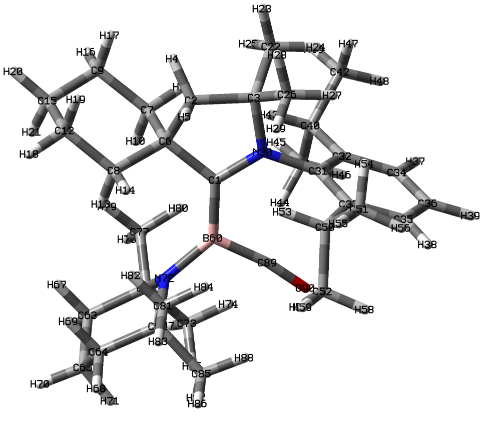

7

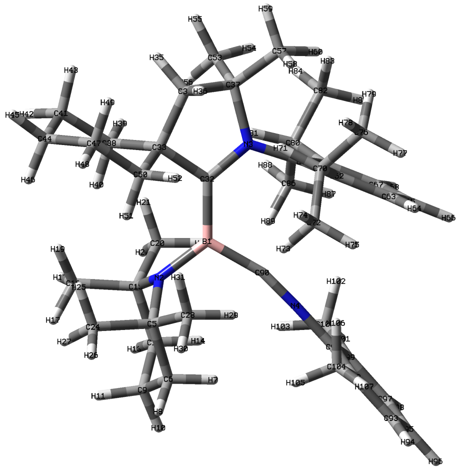 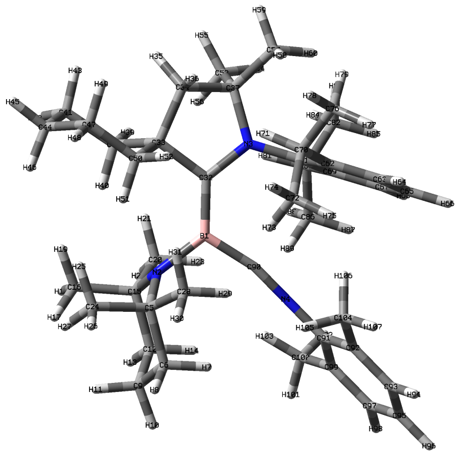

$[\mathbf{9}]^{+\bullet}$ **10**

Supplementary Fig. 30. Optimized structure at UCAM-B3LYP/6-31G**/SMD(CH_2Cl_2) level. Hydrogen atoms are omitted for clarity.

Supplementary Table 8. Cartesian coordinates for the optimized geometry of [**1**]$^{+}$.

Label	Element	X	Y	Z
1	C	-1.10065	0.62481	-0.09404
2	C	0.93221	1.82242	0.30615
3	C	-0.24174	2.80732	0.32441
4	H	1.28153	1.64218	1.32599
5	H	1.76989	2.23387	-0.25674
6	C	0.42031	0.50612	-0.29167
7	C	1.14555	-0.71486	0.30244
8	C	0.59184	0.4511	-1.84678
9	C	2.61073	-0.79834	-0.1361
10	H	0.63059	-1.62892	-0.00008
11	H	1.11775	-0.67274	1.3918
12	C	2.05066	0.32746	-2.29295
13	H	0.0344	-0.40928	-2.23267
14	H	0.15306	1.33927	-2.30122
15	C	2.74524	-0.86848	-1.6528
16	H	3.06173	-1.67908	0.33181
17	H	3.1639	0.07062	0.2411
18	H	2.06665	0.24914	-3.38461
19	H	2.59708	1.24364	-2.04087
20	H	3.79959	-0.89776	-1.94521
21	H	2.28831	-1.79744	-2.01743
22	C	-0.42094	3.42554	1.70552
23	H	0.48545	3.99472	1.92768
24	H	-1.26209	4.11845	1.73147
25	H	-0.53807	2.67166	2.48497
26	C	-0.07879	3.93457	-0.68278
27	H	-0.95738	4.57868	-0.70535
28	H	0.76509	4.5434	-0.34971
29	H	0.14701	3.57974	-1.68677
30	N	-1.44266	1.88232	0.01168
31	C	-2.78345	2.47989	-0.19256
32	C	-3.65955	2.66148	0.89779
33	C	-3.1435	2.92514	-1.49352
34	C	-4.89514	3.26885	0.66275
35	C	-4.39704	3.51491	-1.65243
36	C	-5.27143	3.69055	-0.59583

37	H	-5.57472	3.4053	1.49609
38	H	-4.68963	3.85226	-2.6402
39	H	-6.23812	4.15725	-0.75453
40	C	-3.40911	2.20945	2.32843
41	C	-4.41322	1.11689	2.72221
42	C	-3.49686	3.35973	3.34035
43	H	-2.40448	1.79651	2.38976
44	H	-4.43545	0.29953	1.99835
45	H	-4.16321	0.70056	3.70254
46	H	-5.4253	1.52638	2.78431
47	H	-2.83859	4.19274	3.08735
48	H	-4.51585	3.74958	3.41118
49	H	-3.2164	2.99771	4.33413
50	C	-2.29284	2.86253	-2.76443
51	C	-2.03893	4.2689	-3.33728
52	C	-2.92285	2.01964	-3.8833
53	H	-1.33314	2.41315	-2.52382
54	H	-1.69247	4.98389	-2.59111
55	H	-1.28677	4.21421	-4.13004
56	H	-2.95157	4.67403	-3.7834
57	H	-3.04167	0.97424	-3.60721
58	H	-3.90325	2.40941	-4.17161
59	H	-2.2817	2.06321	-4.7691
60	B	-2.05449	-0.69755	-0.27856
61	Cl	-3.32207	-0.45948	-1.52325
62	C	-1.82352	-2.11694	1.83395
63	C	-2.01038	-3.23384	-0.49615
64	C	-0.80404	-3.18197	2.25456
65	C	-0.9968	-4.26903	0.04446
66	C	-1.01828	-4.50614	1.54296
67	H	-0.8795	-3.30283	3.33983
68	H	0.20543	-2.81413	2.04291
69	H	-1.18365	-5.20187	-0.49634
70	H	0.00896	-3.9369	-0.24026
71	H	-0.22659	-5.21195	1.8128
72	H	-1.96108	-4.96774	1.85273
73	N	-1.88581	-1.95533	0.32923
74	C	-3.22216	-2.49153	2.36578
75	H	-3.98515	-1.82687	1.95591

76	H	-3.51225	-3.5156	2.14423
77	H	-3.22715	-2.38226	3.45403
78	C	-1.46186	-0.80181	2.51441
79	H	-1.42893	-0.9691	3.59366
80	H	-0.48884	-0.41424	2.22356
81	H	-2.22307	-0.04869	2.33343
82	C	-1.56742	-3.00599	-1.95291
83	H	-0.6436	-2.42395	-2.003
84	H	-1.36198	-3.98318	-2.39705
85	H	-2.32153	-2.52692	-2.57269
86	C	-3.4296	-3.82231	-0.51895
87	H	-3.49202	-4.55667	-1.32723
88	H	-3.6946	-4.3373	0.40328
89	H	-4.17629	-3.05037	-0.71178

Supplementary Table 9. Cartesian coordinates for the optimized geometry of **2•**.

Label	Element	X	Y	Z
1	C	-1.1329	0.60701	-0.05849
2	C	0.86772	1.85008	0.40724
3	C	-0.2668	2.85841	0.21751
4	H	1.03775	1.69328	1.47739
5	H	1.80033	2.22693	-0.01526
6	C	0.40789	0.52637	-0.22016
7	C	1.12712	-0.65889	0.44806
8	C	0.73462	0.48302	-1.7424
9	C	2.62886	-0.69793	0.14659
10	H	0.66658	-1.59159	0.11892
11	H	0.98975	-0.60739	1.53042
12	C	2.23025	0.41163	-2.06302
13	H	0.24524	-0.39277	-2.1733
14	H	0.30066	1.34732	-2.24617
15	C	2.903	-0.76002	-1.35335
16	H	3.07021	-1.56473	0.65084
17	H	3.12135	0.18719	0.56907
18	H	2.35669	0.3278	-3.14813
19	H	2.72608	1.34474	-1.769
20	H	3.98082	-0.76061	-1.54963
21	H	2.50849	-1.70329	-1.75406
22	C	-0.42525	3.71172	1.47547
23	H	0.50035	4.27468	1.6276
24	H	-1.23807	4.43347	1.37658
25	H	-0.59134	3.10084	2.36291
26	C	-0.01387	3.82509	-0.94343
27	H	-0.86248	4.49732	-1.08219
28	H	0.85525	4.44112	-0.69696
29	H	0.19412	3.32362	-1.88735
30	N	-1.45319	1.95334	-0.01237
31	C	-2.75634	2.56972	-0.17438
32	C	-3.56365	2.85572	0.94947
33	C	-3.19169	2.93777	-1.4696
34	C	-4.75546	3.55656	0.75642
35	C	-4.39509	3.63008	-1.60215
36	C	-5.16984	3.95529	-0.50195

37	H	-5.37758	3.78092	1.61664
38	H	-4.73324	3.91196	-2.59392
39	H	-6.10041	4.50044	-0.62679
40	C	-3.27528	2.37383	2.36702
41	C	-4.33654	1.35897	2.81928
42	C	-3.22523	3.51494	3.39254
43	H	-2.30915	1.86679	2.36342
44	H	-4.42326	0.5237	2.12314
45	H	-4.08422	0.96154	3.80766
46	H	-5.32006	1.83265	2.89685
47	H	-2.52544	4.30418	3.11432
48	H	-4.21079	3.97345	3.51777
49	H	-2.92317	3.12552	4.37004
50	C	-2.46135	2.56915	-2.75584
51	C	-2.16698	3.78518	-3.64448
52	C	-3.25237	1.53663	-3.57138
53	H	-1.51461	2.10796	-2.48356
54	H	-1.64589	4.58098	-3.10905
55	H	-1.54328	3.48528	-4.4927
56	H	-3.08831	4.20897	-4.05552
57	H	-3.45349	0.63409	-2.99319
58	H	-4.2131	1.94673	-3.89949
59	H	-2.68948	1.25556	-4.46761
60	B	-2.08791	-0.5881	-0.02167
61	Cl	-3.88013	-0.27346	-0.48036
62	C	-1.94199	-2.34663	1.80032
63	C	-1.90528	-3.06783	-0.65034
64	C	-0.97694	-3.49907	2.13087
65	C	-0.93106	-4.19759	-0.26195
66	C	-1.09778	-4.67771	1.17339
67	H	-1.15832	-3.82165	3.16248
68	H	0.04854	-3.11258	2.09269
69	H	-1.0627	-5.02841	-0.96447
70	H	0.09367	-3.82904	-0.39314
71	H	-0.33601	-5.42948	1.40682
72	H	-2.06554	-5.17498	1.30015
73	N	-1.7922	-1.96834	0.35838
74	C	-3.38522	-2.74604	2.18815
75	H	-4.10455	-2.02151	1.80101

76	H	-3.67371	-3.73235	1.82858
77	H	-3.47735	-2.76448	3.27893
78	C	-1.587	-1.16515	2.70931
79	H	-1.61122	-1.50163	3.75
80	H	-0.59375	-0.76925	2.50811
81	H	-2.30799	-0.35232	2.61342
82	C	-1.45514	-2.53983	-2.01569
83	H	-0.42327	-2.19027	-1.97731
84	H	-1.50624	-3.34406	-2.75509
85	H	-2.09012	-1.72494	-2.37512
86	C	-3.31954	-3.6597	-0.84769
87	H	-3.31806	-4.29678	-1.73803
88	H	-3.64646	-4.28065	-0.01505
89	H	-4.05892	-2.87297	-0.99722

Supplementary Table 10. Cartesian coordinates for the optimized geometry of **[3]**$^{+\bullet}$.

Label	Element	X	Y	Z
1	C	-0.97684	0.70971	-0.0427
2	C	0.8148	2.27465	-0.33028
3	C	-0.41829	2.98316	0.24864
4	H	1.71446	2.56286	0.21503
5	H	0.95007	2.58021	-1.37149
6	C	0.54371	0.75271	-0.25953
7	C	1.33749	0.04792	0.86119
8	C	0.87363	0.05544	-1.6008
9	C	2.84143	-0.00564	0.57991
10	H	0.9643	-0.9792	0.9545
11	H	1.15276	0.54015	1.82045
12	C	2.37469	0.03191	-1.89234
13	H	0.50834	-0.97881	-1.55749
14	H	0.33117	0.55278	-2.41157
15	C	3.14282	-0.65989	-0.76759
16	H	3.33761	-0.55127	1.38908
17	H	3.25788	1.0088	0.59099
18	H	2.5501	-0.47584	-2.84636
19	H	2.74491	1.0577	-2.01272
20	H	4.21874	-0.63662	-0.96872
21	H	2.85109	-1.7178	-0.73041
22	C	-0.28721	3.17448	1.76439
23	H	0.60128	3.77777	1.96859
24	H	-1.14824	3.70238	2.17518
25	H	-0.17895	2.22262	2.28792
26	C	-0.67657	4.33441	-0.39767
27	H	-1.62868	4.76328	-0.07624
28	H	0.11623	5.01863	-0.08452
29	H	-0.65607	4.28252	-1.4844
30	N	-1.49575	1.96269	-0.03655
31	C	-2.87543	2.28156	-0.34734
32	C	-3.85208	2.35346	0.66515
33	C	-3.21188	2.52944	-1.69963
34	C	-5.1622	2.67047	0.29964
35	C	-4.53971	2.82643	-2.00409
36	C	-5.51054	2.90087	-1.01893

37	H	-5.92175	2.73545	1.07125
38	H	-4.81317	3.01245	-3.03718
39	H	-6.53624	3.14177	-1.27944
40	B	-1.67188	-0.55546	0.18195
41	C	-3.57525	2.10902	2.13956
42	C	-4.48462	1.00949	2.70376
43	C	-3.75601	3.39339	2.95961
44	H	-2.53997	1.77827	2.24365
45	H	-4.43404	0.09442	2.10839
46	H	-4.19546	0.76621	3.73048
47	H	-5.52912	1.33231	2.72722
48	H	-3.15806	4.22099	2.57028
49	H	-4.80251	3.71289	2.95266
50	H	-3.4667	3.22462	4.00138
51	C	-2.21692	2.51419	-2.85487
52	C	-2.21601	3.84801	-3.61721
53	C	-2.50249	1.37853	-3.84403
54	H	-1.21689	2.35374	-2.44874
55	H	-2.11454	4.71	-2.95459
56	H	-1.38918	3.86904	-4.33358
57	H	-3.1423	3.97608	-4.18457
58	H	-2.46346	0.39972	-3.36679
59	H	-3.49243	1.49141	-4.29657
60	H	-1.76571	1.39108	-4.65322
61	C	-2.80675	-2.59889	-0.647
62	C	-2.00167	-2.37804	1.84011
63	C	-4.12961	-3.15364	-0.10301
64	C	-3.35794	-2.96127	2.26243
65	C	-3.98365	-3.88452	1.22488
66	H	-4.55194	-3.81318	-0.86745
67	H	-4.82878	-2.318	0.02133
68	H	-3.20845	-3.48695	3.21062
69	H	-4.04407	-2.13068	2.46281
70	H	-4.96746	-4.21206	1.57353
71	H	-3.3828	-4.79133	1.10139
72	N	-2.13578	-1.78895	0.44487
73	C	-0.89591	-3.44089	1.88098
74	H	-1.17734	-4.37809	1.40482
75	H	0.01929	-3.07369	1.41009

76	H	-0.66974	-3.66307	2.92719
77	C	-1.61616	-1.2707	2.81993
78	H	-0.60575	-0.89824	2.63997
79	H	-2.31567	-0.43534	2.78221
80	H	-1.64027	-1.68068	3.83266
81	C	-1.88786	-3.7153	-1.15885
82	H	-2.31188	-4.11607	-2.08333
83	H	-0.89255	-3.32525	-1.38705
84	H	-1.78281	-4.54593	-0.46374
85	C	-3.10026	-1.66891	-1.8196
86	H	-3.68622	-0.79795	-1.51238
87	H	-2.17508	-1.33383	-2.29533
88	H	-3.67705	-2.21216	-2.57214

Supplementary Table 11. Cartesian coordinates for the optimized geometry of **4**.

Label	Element	X	Y	Z
1	C	-0.99834	0.65159	-0.01023
2	C	0.75752	2.29296	-0.26988
3	C	-0.49054	2.92868	0.3813
4	H	1.66713	2.54798	0.28027
5	H	0.87239	2.69653	-1.28107
6	C	0.51057	0.75988	-0.33483
7	C	1.38241	-0.00782	0.68274
8	C	0.83435	0.20223	-1.73994
9	C	2.87832	0.00393	0.35321
10	H	1.04008	-1.05062	0.70643
11	H	1.21847	0.39391	1.6877
12	C	2.32394	0.25385	-2.08737
13	H	0.50358	-0.84383	-1.77831
14	H	0.24924	0.74124	-2.49111
15	C	3.15238	-0.51383	-1.05831
16	H	3.42042	-0.59806	1.09135
17	H	3.27065	1.02471	0.44299
18	H	2.48638	-0.15612	-3.09075
19	H	2.66205	1.2978	-2.12112
20	H	4.22075	-0.44222	-1.29098
21	H	2.88945	-1.57909	-1.10992
22	C	-0.32252	2.97375	1.91113
23	H	0.58732	3.51938	2.18247
24	H	-1.16619	3.47908	2.38662
25	H	-0.24998	1.96623	2.32753
26	C	-0.73427	4.34713	-0.11987
27	H	-1.66391	4.76075	0.28176
28	H	0.08419	4.99447	0.20906
29	H	-0.77872	4.39276	-1.2084
30	N	-1.54049	1.97841	-0.02996
31	C	-2.86535	2.31029	-0.44245
32	C	-3.9249	2.35921	0.49323
33	C	-3.14119	2.59144	-1.80557
34	C	-5.21243	2.68694	0.06188
35	C	-4.44581	2.89956	-2.19177
36	C	-5.47966	2.95294	-1.27021

37	H	-6.01992	2.73153	0.78646
38	H	-4.65325	3.10828	-3.23702
39	H	-6.48746	3.20164	-1.58938
40	B	-1.62966	-0.55865	0.33616
41	C	-3.72149	2.07121	1.97122
42	C	-4.64942	0.95553	2.46653
43	C	-3.92419	3.33307	2.81949
44	H	-2.69144	1.73373	2.09645
45	H	-4.52961	0.0449	1.87379
46	H	-4.43199	0.71249	3.5116
47	H	-5.70129	1.25314	2.41429
48	H	-3.28722	4.15628	2.48456
49	H	-4.96215	3.67813	2.76764
50	H	-3.69373	3.13315	3.87113
51	C	-2.07054	2.57379	-2.88517
52	C	-1.99961	3.8998	-3.6542
53	C	-2.28076	1.42028	-3.87262
54	H	-1.11414	2.41807	-2.38597
55	H	-1.89994	4.75935	-2.98624
56	H	-1.14065	3.89813	-4.3331
57	H	-2.89542	4.05778	-4.26285
58	H	-2.29547	0.45404	-3.36523
59	H	-3.22778	1.53049	-4.41145
60	H	-1.47779	1.40063	-4.61704
61	C	-2.83842	-2.60353	-0.45516
62	C	-1.9875	-2.39765	1.99267
63	C	-4.10735	-3.26582	0.10648
64	C	-3.29633	-3.0673	2.44302
65	C	-3.88264	-4.01702	1.40953
66	H	-4.50952	-3.93179	-0.66409
67	H	-4.85818	-2.48383	0.27397
68	H	-3.10226	-3.58886	3.38606
69	H	-4.0303	-2.28149	2.65902
70	H	-4.8315	-4.42434	1.77317
71	H	-3.21901	-4.87395	1.25163
72	N	-2.16128	-1.78963	0.62047
73	C	-0.82836	-3.40738	2.01068
74	H	-1.04923	-4.33226	1.4796
75	H	0.07003	-2.96656	1.57103

76	H	-0.60002	-3.67435	3.0468
77	C	-1.64943	-1.30514	3.00873
78	H	-0.66402	-0.87176	2.83058
79	H	-2.38685	-0.50117	2.9939
80	H	-1.64803	-1.74882	4.00881
81	C	-1.88796	-3.65523	-1.04912
82	H	-2.32862	-4.0663	-1.96221
83	H	-0.93043	-3.19814	-1.31364
84	H	-1.69386	-4.49193	-0.37885
85	C	-3.26501	-1.6796	-1.59541
86	H	-3.87257	-0.84724	-1.23135
87	H	-2.40199	-1.27179	-2.12268
88	H	-3.86151	-2.252	-2.31167

Supplementary Table 12. Cartesian coordinates for the optimized geometry of **7**.

Label	Element	X	Y	Z
1	C	-1.00708	0.72804	-0.05741
2	C	0.93582	2.0552	-0.58215
3	C	-0.24481	3.00972	-0.39547
4	H	1.85011	2.47214	-0.15807
5	H	1.11271	1.90731	-1.65237
6	C	0.53228	0.71234	0.05083
7	C	0.92707	0.66319	1.55268
8	C	1.22132	-0.45763	-0.67021
9	C	2.44011	0.62285	1.78527
10	H	0.48825	-0.23078	1.99222
11	H	0.49244	1.51103	2.08665
12	C	2.73693	-0.47707	-0.45331
13	H	0.78738	-1.39619	-0.31651
14	H	1.00831	-0.39041	-1.74058
15	C	3.09211	-0.53422	1.03077
16	H	2.63089	0.53345	2.8604
17	H	2.90139	1.56667	1.47037
18	H	3.16478	-1.33682	-0.98042
19	H	3.19081	0.4162	-0.90137
20	H	4.17891	-0.51593	1.16743
21	H	2.7373	-1.48534	1.44928
22	C	-0.07423	3.95444	0.79848
23	H	0.78486	4.60484	0.61217
24	H	-0.9513	4.59496	0.90993
25	H	0.09906	3.43435	1.73948
26	C	-0.42635	3.88636	-1.63292
27	H	-1.30364	4.53155	-1.54787
28	H	0.45048	4.53303	-1.73027
29	H	-0.50383	3.29326	-2.54367
30	N	-1.37366	2.03508	-0.21089
31	C	-2.73774	2.49262	-0.16298
32	C	-3.31724	2.8216	1.08614
33	C	-3.49078	2.61454	-1.35484
34	C	-4.61692	3.32604	1.10554
35	C	-4.78601	3.12568	-1.27225
36	C	-5.34571	3.4915	-0.06003

37	H	-5.06878	3.58398	2.05773
38	H	-5.37145	3.22683	-2.18014
39	H	-6.35529	3.889	-0.02195
40	C	-2.63452	2.59437	2.42974
41	C	-3.36366	1.51535	3.2437
42	C	-2.54258	3.87564	3.26871
43	H	-1.62373	2.23422	2.24081
44	H	-3.44135	0.5758	2.69438
45	H	-2.82335	1.31925	4.17545
46	H	-4.37559	1.83677	3.5096
47	H	-2.06416	4.69562	2.72983
48	H	-3.53466	4.21656	3.58006
49	H	-1.96365	3.68674	4.17824
50	C	-3.01055	2.15481	-2.72675
51	C	-3.02059	3.28662	-3.76387
52	C	-3.87594	1.00071	-3.25361
53	H	-1.98986	1.78231	-2.62236
54	H	-2.46038	4.16373	-3.43694
55	H	-2.58349	2.93714	-4.7046
56	H	-4.04374	3.60827	-3.98089
57	H	-3.91357	0.16665	-2.55238
58	H	-4.90242	1.33125	-3.43976
59	H	-3.47195	0.62959	-4.20085
60	B	-1.96295	-0.45325	0.02984
61	C	-1.94005	-2.74695	1.25857
62	C	-2.0111	-2.66663	-1.295
63	C	-1.27906	-4.13594	1.14568
64	C	-1.3377	-4.05342	-1.31124
65	C	-1.67825	-4.9051	-0.10073
66	H	-1.51274	-4.70498	2.05231
67	H	-0.19064	-3.99684	1.13046
68	H	-1.6159	-4.56212	-2.24103
69	H	-0.25024	-3.90945	-1.34084
70	H	-1.14439	-5.86088	-0.14499
71	H	-2.74644	-5.14817	-0.08271
72	N	-1.72997	-1.9592	0.00073
73	C	-3.41788	-2.92423	1.67553
74	H	-3.91048	-1.95978	1.81326
75	H	-4.00399	-3.49861	0.95935

76	H	-3.46829	-3.45301	2.63298
77	C	-1.24455	-2.05282	2.43156
78	H	-1.41881	-2.62086	3.35056
79	H	-0.16625	-2.01033	2.26954
80	H	-1.6178	-1.03871	2.58711
81	C	-1.40308	-1.88865	-2.46784
82	H	-0.32127	-1.80831	-2.3687
83	H	-1.61468	-2.41918	-3.40131
84	H	-1.81615	-0.88295	-2.55284
85	C	-3.51203	-2.84768	-1.62216
86	H	-3.62171	-3.22417	-2.64442
87	H	-4.01251	-3.55927	-0.96607
88	H	-4.05494	-1.90312	-1.56479
89	C	-3.40617	-0.12977	0.10015
90	O	-4.56672	-0.07385	0.19939

Supplementary Table 13. Cartesian coordinates for the optimized geometry of [**8**]$^{+\bullet}$.

Label	Element	X	Y	Z
1	C	-1.02152	0.73507	-0.02988
2	C	0.9128	2.14524	-0.31706
3	C	-0.2254	2.94563	0.30973
4	H	1.85176	2.34567	0.19862
5	H	1.03867	2.45097	-1.35937
6	C	0.50293	0.65363	-0.24888
7	C	1.32609	-0.13552	0.7924
8	C	0.71723	-0.03321	-1.6236
9	C	2.80748	-0.24082	0.4143
10	H	0.91919	-1.14682	0.86308
11	H	1.23951	0.32716	1.77872
12	C	2.19421	-0.09742	-2.01609
13	H	0.33668	-1.05524	-1.56038
14	H	0.13879	0.48697	-2.39372
15	C	3.00369	-0.85726	-0.96856
16	H	3.31652	-0.84062	1.1755
17	H	3.27513	0.75012	0.44056
18	H	2.27782	-0.58332	-2.99342
19	H	2.5996	0.9143	-2.13629
20	H	4.06552	-0.8645	-1.2344
21	H	2.67382	-1.90411	-0.94952
22	C	-0.03754	3.10845	1.82059
23	H	0.89308	3.65516	1.99213
24	H	-0.84745	3.68526	2.26697
25	H	0.03409	2.14612	2.33197
26	C	-0.41801	4.3149	-0.32041
27	H	-1.30273	4.82285	0.06955
28	H	0.45289	4.9237	-0.06451
29	H	-0.47946	4.26892	-1.40625
30	N	-1.38957	1.99523	0.05544
31	C	-2.74421	2.47237	-0.18003
32	C	-3.65489	2.6518	0.8793
33	C	-3.09232	2.76703	-1.51976
34	C	-4.93115	3.11782	0.56427
35	C	-4.38622	3.2196	-1.76709
36	C	-5.29984	3.39527	-0.7407

37	H	-5.65074	3.25689	1.36339
38	H	-4.68239	3.43698	-2.78706
39	H	-6.30179	3.75009	-0.9592
40	C	-3.35554	2.33349	2.33468
41	C	-4.32221	1.27258	2.87788
42	C	-3.42405	3.59281	3.20812
43	H	-2.34454	1.92785	2.39846
44	H	-4.34316	0.38096	2.24627
45	H	-4.02448	0.97011	3.88614
46	H	-5.34275	1.66109	2.9379
47	H	-2.77979	4.39252	2.83396
48	H	-4.445	3.98387	3.24832
49	H	-3.11774	3.35966	4.23217
50	C	-2.15817	2.59406	-2.71336
51	C	-2.09327	3.86072	-3.57818
52	C	-2.56941	1.40225	-3.58743
53	H	-1.1514	2.39459	-2.3449
54	H	-1.8884	4.75694	-2.98776
55	H	-1.30162	3.7571	-4.32594
56	H	-3.03043	4.02435	-4.11736
57	H	-2.51446	0.45478	-3.04743
58	H	-3.59158	1.52088	-3.95933
59	H	-1.90522	1.32867	-4.45389
60	B	-2.06935	-0.45755	-0.08844
61	C	-1.87014	-2.26534	1.72566
62	C	-2.30002	-2.94174	-0.71565
63	C	-0.86408	-3.41974	1.85975
64	C	-1.25937	-4.06369	-0.52863
65	C	-1.14825	-4.57175	0.90331
66	H	-0.87383	-3.77053	2.89721
67	H	0.14193	-3.03115	1.66473
68	H	-1.51726	-4.88433	-1.20657
69	H	-0.28387	-3.67812	-0.8497
70	H	-0.34606	-5.31375	0.96892
71	H	-2.06676	-5.09164	1.19484
72	N	-1.97639	-1.8573	0.28146
73	C	-3.23835	-2.67628	2.31224
74	H	-4.0121	-1.95535	2.03615
75	H	-3.56997	-3.66561	2.00659

76	H	-3.16683	-2.68649	3.4036
77	C	-1.40183	-1.09275	2.58736
78	H	-1.27914	-1.44408	3.61515
79	H	-0.44762	-0.68766	2.26885
80	H	-2.1417	-0.29065	2.60572
81	C	-2.15076	-2.41876	-2.15099
82	H	-1.20923	-1.89283	-2.3043
83	H	-2.16752	-3.27383	-2.83188
84	H	-2.97191	-1.76397	-2.45116
85	C	-3.72996	-3.51511	-0.61835
86	H	-3.95756	-4.04817	-1.54581
87	H	-3.86253	-4.2259	0.19443
88	H	-4.47578	-2.72548	-0.50365
89	C	-3.4667	-0.11224	-0.57385
90	O	-4.55405	-0.10525	-0.9551

Supplementary Table 14. Cartesian coordinates for the optimized geometry of [**9**]$^{+\bullet}$.

Label	Element	X	Y	Z
1	B	12.64953	16.30269	14.24301
2	N	11.44665	17.16537	14.20699
3	N	15.19319	16.01946	14.63011
4	N	11.98423	14.05163	12.87215
5	C	10.17759	16.7143	14.87926
6	C	9.15439	16.17005	13.86049
7	H	9.5156	15.20625	13.48248
8	H	8.21377	15.96756	14.38449
9	C	8.92058	17.10027	12.67835
10	H	8.25866	16.61958	11.9504
11	H	8.4058	18.0107	13.00299
12	C	10.25099	17.44341	12.02247
13	H	10.10303	18.14224	11.19149
14	H	10.6808	16.52886	11.59477
15	C	11.2801	18.05224	12.99799
16	C	10.85452	19.49399	13.34613
17	H	9.79277	19.58918	13.5675
18	H	11.05692	20.14199	12.48806
19	H	11.41136	19.88652	14.19804
20	C	12.60676	18.15845	12.23826
21	H	13.38228	18.65624	12.82257
22	H	12.44892	18.74342	11.32798
23	H	12.97727	17.17797	11.93362
24	C	9.53694	17.85486	15.69533
25	H	10.27033	18.32631	16.35363
26	H	8.73936	17.44458	16.32219
27	H	9.08986	18.63212	15.07926
28	C	10.46433	15.58914	15.88029
29	H	10.85387	14.69266	15.40005
30	H	9.52695	15.3041	16.36621
31	H	11.15838	15.89756	16.66298
32	C	14.02615	16.63479	14.87704
33	C	14.25564	17.72634	15.94123
34	C	15.68926	17.4243	16.42554
35	H	16.24074	18.3176	16.71731
36	H	15.64462	16.76535	17.29833

37	C	16.38977	16.69419	15.28506
38	C	14.09286	19.1624	15.3904
39	H	14.78566	19.34416	14.56562
40	H	13.08527	19.24296	14.98201
41	C	14.27358	20.2395	16.46447
42	H	14.08918	21.22035	16.01355
43	H	15.31221	20.25519	16.8151
44	C	13.33954	20.01975	17.65367
45	H	13.53896	20.75984	18.4357
46	H	12.30178	20.17408	17.33058
47	C	13.48312	18.6032	18.20805
48	H	12.76086	18.43492	19.01377
49	H	14.47804	18.47921	18.653
50	C	13.26196	17.55983	17.11075
51	H	12.24877	17.67168	16.72372
52	H	13.34762	16.54893	17.52415
53	C	17.07538	17.68463	14.33464
54	H	17.68012	17.17791	13.58499
55	H	17.74848	18.30845	14.92835
56	H	16.36869	18.34293	13.82931
57	C	17.43658	15.702	15.77263
58	H	17.06347	15.0592	16.56787
59	H	18.27842	16.27097	16.17583
60	H	17.81881	15.08093	14.95898
61	C	15.29222	14.75448	13.92759
62	C	15.05598	13.57603	14.67259
63	C	15.0551	12.35908	13.99277
64	H	14.85926	11.44656	14.54613
65	C	15.31319	12.29257	12.63292
66	H	15.307	11.33485	12.12248
67	C	15.6113	13.45096	11.93404
68	H	15.85803	13.388	10.8797
69	C	15.61563	14.70072	12.55617
70	C	14.85066	13.549	16.18407
71	H	14.98781	14.56009	16.56863
72	C	13.44337	13.10155	16.58398
73	H	12.67792	13.75169	16.15888
74	H	13.33776	13.12622	17.67304
75	H	13.24693	12.07572	16.25848

76	C	15.88549	12.64056	16.86377
77	H	15.70699	11.58885	16.62207
78	H	15.81592	12.74305	17.95094
79	H	16.90812	12.88045	16.56412
80	C	16.05638	15.9043	11.73482
81	H	15.88966	16.80159	12.32948
82	C	17.55708	15.78961	11.42222
83	H	18.15489	15.58252	12.31315
84	H	17.92624	16.71428	10.96821
85	H	17.73637	14.97547	10.71302
86	C	15.29091	16.08639	10.42102
87	H	15.44348	15.24407	9.74023
88	H	15.65366	16.98434	9.91155
89	H	14.21887	16.20647	10.57844
90	C	12.43361	14.94976	13.46223
91	C	11.39551	13.00983	12.15348
92	C	10.70132	12.01055	12.85455
93	C	10.11742	10.99404	12.10365
94	H	9.57969	10.20724	12.62241
95	C	10.21322	10.97804	10.7193
96	H	9.74913	10.17589	10.15495
97	C	10.89999	11.98468	10.05362
98	H	10.97317	11.97261	8.97124
99	C	11.50646	13.02261	10.75318
100	C	12.25723	14.10213	10.03609
101	H	12.21059	13.94718	8.95709
102	H	13.30697	14.10785	10.33629
103	H	11.8461	15.09195	10.25595
104	C	10.56382	12.03099	14.34707
105	H	9.83343	12.78201	14.66349
106	H	11.50941	12.26526	14.83725
107	H	10.22057	11.06039	14.70876

Supplementary Table 15. Cartesian coordinates for the optimized geometry of **10**.

Label	Element	X	Y	Z
1	B	12.68927	16.35726	14.15619
2	N	11.42409	17.21762	14.18093
3	N	15.18908	15.88744	14.71544
4	N	12.13021	14.3771	12.42691
5	C	10.19767	16.72679	14.89091
6	C	9.12419	16.20773	13.9066
7	H	9.49334	15.27993	13.45207
8	H	8.22189	15.94372	14.47105
9	C	8.79304	17.19431	12.79751
10	H	8.08852	16.74436	12.08882
11	H	8.28978	18.07733	13.20673
12	C	10.07483	17.59096	12.08043
13	H	9.86762	18.33612	11.303
14	H	10.47391	16.70651	11.57176
15	C	11.1679	18.14294	13.02499
16	C	10.75036	19.56719	13.46765
17	H	9.71704	19.63087	13.80494
18	H	10.84671	20.25645	12.62247
19	H	11.3861	19.93882	14.27323
20	C	12.42682	18.32773	12.16463
21	H	13.24839	18.77588	12.7249
22	H	12.18505	18.99526	11.33125
23	H	12.78447	17.38668	11.74567
24	C	9.56148	17.80786	15.79616
25	H	10.31543	18.28207	16.42835
26	H	8.81733	17.34602	16.45344
27	H	9.04864	18.59333	15.2442
28	C	10.52855	15.55346	15.81928
29	H	10.92633	14.7027	15.26758
30	H	9.61105	15.2271	16.31942
31	H	11.25099	15.82596	16.58906
32	C	13.98952	16.59632	14.84819
33	C	14.26112	17.71709	15.88784
34	C	15.70111	17.43445	16.36851
35	H	16.26702	18.34093	16.59036
36	H	15.67133	16.83964	17.28757

37	C	16.37017	16.61096	15.27087
38	C	14.1148	19.14144	15.30636
39	H	14.80969	19.29097	14.47531
40	H	13.10905	19.21865	14.89357
41	C	14.30778	20.25428	16.34223
42	H	14.13015	21.2243	15.86396
43	H	15.34772	20.27318	16.69076
44	C	13.37888	20.08033	17.54325
45	H	13.58295	20.84743	18.29871
46	H	12.33976	20.22608	17.21973
47	C	13.52094	18.68225	18.14352
48	H	12.80707	18.54706	18.96403
49	H	14.52064	18.57243	18.58311
50	C	13.2898	17.60348	17.08241
51	H	12.27078	17.69523	16.70547
52	H	13.38015	16.60693	17.52928
53	C	17.04711	17.53297	14.24045
54	H	17.6341	16.96666	13.51727
55	H	17.73349	18.21098	14.75717
56	H	16.32315	18.13961	13.69424
57	C	17.4395	15.67943	15.83291
58	H	17.06258	15.07094	16.65421
59	H	18.26547	16.28402	16.21895
60	H	17.84779	15.01714	15.06483
61	C	15.28717	14.56537	14.17385
62	C	15.07009	13.4586	15.0323
63	C	15.16395	12.16984	14.50797
64	H	14.99726	11.31884	15.16088
65	C	15.47451	11.953	13.1738
66	H	15.54344	10.94148	12.78514
67	C	15.71092	13.03706	12.34618
68	H	15.97308	12.86648	11.30672
69	C	15.62786	14.34813	12.82061
70	C	14.76004	13.60349	16.51708
71	H	14.85926	14.65937	16.77208
72	C	13.32423	13.18524	16.84942
73	H	12.60235	13.76068	16.27051
74	H	13.1177	13.35042	17.9122
75	H	13.16286	12.12194	16.6432

76	C	15.73826	12.80483	17.38969
77	H	15.58754	11.72713	17.27404
78	H	15.57965	13.0429	18.44633
79	H	16.78231	13.01909	17.14811
80	C	15.95044	15.47482	11.85118
81	H	15.78505	16.41602	12.37389
82	C	17.41775	15.40762	11.40424
83	H	18.10693	15.3356	12.2496
84	H	17.68384	16.29668	10.82338
85	H	17.5903	14.53404	10.76728
86	C	15.04348	15.47406	10.61788
87	H	15.1613	14.55877	10.02906
88	H	15.29387	16.31817	9.96694
89	H	13.99472	15.56327	10.89717
90	C	12.50619	15.17374	13.21647
91	C	11.58883	13.3093	11.72228
92	C	11.28233	12.11902	12.41202
93	C	10.74964	11.05932	11.68643
94	H	10.51452	10.13559	12.20583
95	C	10.5073	11.1739	10.32294
96	H	10.0852	10.338	9.77428
97	C	10.79696	12.35987	9.66678
98	H	10.5991	12.45528	8.60339
99	C	11.34539	13.44805	10.345
100	C	11.65998	14.70441	9.58195
101	H	10.86836	14.91573	8.85861
102	H	12.5923	14.59199	9.01952
103	H	11.76921	15.57079	10.23246
104	C	11.50573	11.99993	13.88908
105	H	10.81771	12.64465	14.44461
106	H	12.5198	12.29442	14.16358
107	H	11.34273	10.97218	14.21964

2. Supplementary References

1 Melaimi, M., Jazzar, R., Soleilhavoup, M. & Bertrand, G. Cyclic (Alkyl)(amino)carbenes (CAACs): Recent Developments. *Angewandte Chemie International Edition* **56**, 10046-10068, doi:https://doi.org/10.1002/anie.201702148 (2017).

2 Nöth, H. & Weber, S. Beiträge zur Chemie des Bors, 137 [1]. Über eine einfache Synthese eines Amino-imino-borans und seiner Addukte mit Bortrichlorid und Bortribromid / Contribution to the Chemistry of Boron, 137 [1]. A Convenient Synthesis of an Amino-Imino-Borane and its Adducts with Boron Trichloride and Boron Tribromide. *Zeitschrift für Naturforschung B* **38**, 1460-1465, doi:doi:10.1515/znb-1983-1123 (1983).

3 Romanato, P., Duttwyler, S., Linden, A., Baldridge, K. K. & Siegel, J. S. Intramolecular Halogen Stabilization of Silylium Ions Directs Gearing Dynamics. *Journal of the American Chemical Society* **132**, 7828-7829, doi:10.1021/ja9109665 (2010).

4 Stoll, S. & Schweiger, A. EasySpin, a comprehensive software package for spectral simulation and analysis in EPR. *Journal of Magnetic Resonance* **178**, 42-55, doi:https://doi.org/10.1016/j.jmr.2005.08.013 (2006).

5 Frisch, M. J. *et al.* Gaussian 16 Rev. C.01. (2016).

6 Dennington, R., Keith, T. A. & Millam, J. M. GaussView, Version 6. (2016).

7 Yan, L., Lu, Y. & Li, X. A density functional theory protocol for the calculation of redox potentials of copper complexes. *Physical Chemistry Chemical Physics* **18**, 5529-5536, doi:10.1039/C5CP06638G (2016).

8 Clark, E. R., Del Grosso, A. & Ingleson, M. J. The Hydride-Ion Affinity of Borenium Cations and Their Propensity to Activate H2 in Frustrated Lewis Pairs. *Chemistry – A European Journal* **19**, 2462-2466, doi:https://doi.org/10.1002/chem.201203318 (2013).

9 Kelly, C. P., Cramer, C. J. & Truhlar, D. G. Aqueous Solvation Free Energies of Ions and Ion−Water Clusters Based on an Accurate Value for the Absolute Aqueous Solvation Free Energy of the Proton. *The Journal of Physical Chemistry B* **110**, 16066-16081, doi:10.1021/jp063552y (2006).
